# Presence of Differences in the Radiofrequency Parameters Applied to Complex Pressure Ulcers: A Secondary Analysis

**DOI:** 10.3390/medicina59030516

**Published:** 2023-03-07

**Authors:** Miguel Ángel Barbas-Monjo, Eleuterio A. Sánchez-Romero, Jorge Hugo Villafañe, Lidia Martínez-Rolando, Jara Velasco García Cuevas, Juan Nicolás Cuenca-Zaldivar

**Affiliations:** 1 Functional Recovery Unit, Guadarrama Hospital, 28440 Madrid, Spain; 2 Department of Physiotherapy, Faculty of Sport Sciences, Universidad Europea de Madrid, 28670 Villaviciosa de Odón, Spain; 3 Physiotherapy and Orofacial Pain Working Group, Sociedad Española de Disfunción Craneomandibular y Dolor Orofacial (SEDCYDO), 28009 Madrid, Spain; 4 Musculoskeletal Pain and Motor Control Research Group, Faculty of Sport Sciences, Universidad Europea de Madrid, 28670 Villaviciosa de Odón, Spain; 5 Department of Physiotherapy, Faculty of Health Sciences, Universidad Europea de Canarias, 38300 Santa Cruz de Tenerife, Spain; 6 Musculoskeletal Pain and Motor Control Research Group, Faculty of Health Sciences, Universidad Europea de Canarias, 38300 Santa Cruz de Tenerife, Spain; 7 IRCCS Fondazione Don Carlo Gnocchi, 20148 Milan, Italy; 8 Rey Juan Carlos University Hospital of Móstoles, 28933 Madrid, Spain; 9 Departamento de Enfermería y Fisioterapia, Grupo de Investigación en Fisioterapia y Dolor, Universidad de Alcalá, Facultad de Medicina y Ciencias de la Salud, 28801 Alcalá de Henares, Spain; 10 Research Group in Nursing and Health Care, Puerta de Hierro Health Research Institute—Segovia de Arana (IDIPHISA), 28222 Majadahonda, Spain; 11 Primary Health Center “El Abajón”, Las Rozas de Madrid, 28231 Madrid, Spain

**Keywords:** pressure ulcers, radiofrequency, expectations

## Abstract

*Background*: Pressure ulcers are a public health problem given the impact that they have on morbidity, mortality and the quality of life and participation of patients who suffer from them. Therefore, the main objective of this study was to evaluate the presence of differences in the radiofrequency parameters applied to complex pressure ulcers throughout the sessions and between the right and left leg. As a secondary objective, the subjective perceptions of the effects of the treatment by both the patients and the practitioner were analyzed. *Methods*: We performed a secondary analysis of data from a prospective study involving 36 patients from the Hospital de Guadarrama in Madrid, Spain, who presented ulcers in the lower limbs. Ten treatment sessions of radiofrequency were administered with a frequency of one session/week, collecting the data referring to the variables in each of the sessions. The main outcome variables were the radiofrequency parameters automatically adjusted in each session and that referred to the frequency (Hz), maximum and average power (W), absorbed energy by the ulcer (J/cm2) and temperature (°C) reached by the tissues. On the other hand, the subjective perception of the results was evaluated using the Global Response Assessment (GRA), a Likert-type scale that scores the treatment results from 1 (significantly worse) to 5 (significantly better). Likewise, the satisfaction of both the patients and the professional were evaluated using a 10-point numerical scale. *Results*: The ANOVA test showed significant differences (*p* < 0.05) throughout the sessions except in patient satisfaction. The ANOVA test showed significant differences (*p* < 0.05) between both legs and over time in all parameters except for frequency. The presence of significant differences (*p* < 0.05) was observed over time between legs compared to the initial values in the absorbed energy and in temperature, with higher final values in the absorbed energy in the left leg compared to the right (26.31 ± 3.75 W vs. 17.36 ± 5.66 W) and a moderate effect on both (R^2^ = 0.471 and 0.492, respectively). The near absence of changes in the satisfaction of both the patients and the professional was observed, while the score in the GRA decreased continuously throughout the sessions. *Conclusions*: Radiofrequency parameters are indicative of an improved clinical response to ulcers. In addition, higher radiofrequency exposure increases healing capacity. However, the subjective perception of treatment outcomes worsened, which may be related to the chronic nature of the ulcers, leading to patients’ expectations not being met.

## 1. Introduction

Pressure ulcers are a public health problem given the impact that they have on morbidity, mortality and the quality of life and participation of patients who suffer with them [1]. The prevalence in Spain is around 7.9% in adults, whereby 65.6% of which are of nosocomial origin; in the United States, they are suffered by some 2.5 million individuals, with percentages ranging between 5% and 15% [1,2]. The classification of these injuries is based on tissue damage from level I with superficial red areas to level IV with significant skin damage that may involve bone, tendon or joint capsule [3]. These hospital-acquired pressure injuries (HAPIs) can lead to chronic wounds, contractures, osteomyelitis, loss of limbs and sepsis, and cause about 60,000 deaths per year [2].

The annual cost of this type of injury is between USD 3.3 billion and USD 11 billion per year and can be as high as USD 26.8 billion in the most advanced stages of HAPI, making the cost per patient about USD 10,708, and each hospital episode costs between USD 500 and more than USD 70,000 in the United States [2].

In diabetic patients, the prevalence of distal ulcers in the lower limbs is between 19% and 34%, being one of the main complications of this pathology. These lesions account for one third of the diabetic patient’s expenses, reaching a total annual expenditure of USD 176 billion; however, despite this high cost, 20% of patients continue to have ulcers after one year of treatment [4].

Given the impact on people’s lives and the cost that pressure ulcers and their complications represent for the healthcare system, prevention should be the most efficient method for dealing with them [1], considering taking measures to address risk factors such as limited movement, the elderly and prolonged embedding [5]. However, the high prevalence makes it necessary to develop and implement treatment methods that limit the process by accelerating recovery and avoiding the appearance of the complications.

In healthy subjects without affectation of the sensory, motor and mental areas, the maintenance of static positions leads to posture modification; however, this does not happen in those patients with alteration of any of the mentioned spheres, causing pressures higher than the filling pressure of the arterial capillaries and higher than the outflow pressure of the venous capillaries to produce tissue hypoxia, resulting in ischemia, tissue damage and subsequent necrosis [6,7].

These lesions can take decades to heal or even fail to heal, which can have an impact on people’s lives, leading to the appearance of secondary diseases such as depression or family distress [8]; however, there are no studies on electrotherapy that analyze factors such as quality of life, depression or the perceived effectiveness of the treatment [3]. Although, it has been evidenced that patients understand that the improvement in the lesion is due to a collaborative approach where they feel more knowledgeable and empowered with tools to improve ulcer care [9].

The use of electrotherapy to address these lesions has been widely studied, especially using electrical stimulation with involvement in the four phases of healing (inflammatory, proliferative, epithelialization and remodeling phase); although the mechanism of action is not well understood, the evidence suggests that this practice increases the flow of blood and thus that of cells, promotes oxygenation, reduces edema and influences dermal growth factors and their receptors [3,10].

Although not as widely studied, radiofrequency has also been used as part of the treatment of HAPI; it began to be used in 1950 and continues to be used today with the aim of improving the remodeling of the injured tissue without causing damage to the surrounding healthy tissue [11].

According to the literature related to the use of radiofrequency in the treatment of chronic pressure ulcers, several studies reflect the use of pulsed radiofrequency as part of the multimodal treatment of these difficult lesions [10,12,13]. These show that this type of treatment, being non-invasive, relatively inexpensive, easy and safe to use and with good patient acceptance is an interesting tool to use as an adjuvant treatment [10]. In relation to multimodal therapy, radiofrequency is combined both with conventional therapy (care appropriate to the lesion) and with negative pressure devices and/or dermal replacement [10,12,13]. The evidence shows the usefulness of pulsed radiofrequency as part of the intervention, achieving progressive healing and thus avoiding amputation of the affected limb [12,13].

Other authors mention a variant of pulsed radiofrequency called pulse dose radiofrequency, where the constant is determined by the voltage and not by the time, as in pulsed radiofrequency, which ensures that the tissue temperature is maintained at 42 °C and allows standardizing a treatment method in relation to the dose and not the exposure time, where preliminary results in small samples show a better effectiveness in reducing pain and maintaining the results achieved [14].

Tecartherapy, included in this group, consists of endogenous thermotherapy using electric current through monopolar capacitive and resistive radiofrequency with the aim of producing heat in the most superficial tissues (capacitive electrode) and in the deepest ones (resistive electrode) [11]. The heat produced in the tissues generates a tissue damage that stimulates fibroblasts and growth factors favoring the production and remodeling of collagen and elastin and the deposition of hyaluronic acid de novo, obtaining as a final result a thickening of the subcutaneous tissue layer that avoids necrosis, fibrosis and damage to vascular and adnexal structures [15].

The main objective of this study was to evaluate the presence of differences between the radiofrequency parameters applied to complex pressure ulcers throughout the sessions and between the right and left leg. As a secondary objective, the subjective perception of the effects of the treatment by both the patients and the practitioner was analyzed.

## 2. Materials and Methods

### 2.1. Study Design

We performed a secondary analysis of data from a prospective study. The previous study aimed to evaluate the effect that the application of radiofrequency at low intensity (frequency) and with non-thermal effects has on the different components of the mechanism of the healing process of hard-to-heal lesions. The methods and description of the study have been previously described [16]. The current study was approved by the Clinical Research Ethics Committee of the Puerta de Hierro Hospital, Madrid, Spain (approval number: 02.18, 6 February 2018). All patients provided informed consent prior to their enrollment. The most relevant parts of the design are summarized below.

### 2.2. Study Population

The study included 36 patients from the Hospital de Guadarrama in Madrid, Spain, who presented ulcers in the lower limbs considering the following aspects of their case histories: age, height and weight, and 13 men and 10 women had diabetes mellitus. These patients came directly from the hospital admission service for ulcer consultation, where they were evaluated by a geriatrician with 20 years of experience (J.V.G.C.). The research team took into account the following inclusion criteria: male or female over or equal to 18 years old or under or equal to 90 years old with a diagnosis of a long-lasting complex wound, that admission was the first one for treatment at Hospital de Guadarrama and that the patient understood and voluntarily signed the corresponding informed consent sheet and information sheet prior to the performance of any evaluation or procedure related to the study. Patients with the following comorbidities were excluded from the study: cardiac pacemaker wearers, presence of local metallic implants, lesion infection, patients with cognitive impairment and patients with malnutrition or risk of malnutrition.

### 2.3. Outcome Measures

The main outcome variables were the radiofrequency parameters automatically adjusted in each session and that referred to the frequency (Hz), maximum and average power (W), absorbed energy by the ulcer (J/cm^2^) and temperature (ºC) reached by the tissues.

On the other hand, the subjective perception of the results was evaluated using the Global Response Assessment (GRA), a Likert-type scale that scores the treatment results from 1 (significantly worse) to 5 (significantly better) [11,17]. Likewise, the satisfaction of both the patients and the professional were evaluated using a 10-point numerical scale.

Ten treatment sessions were administered with a frequency of one session/week, with all outcome measures measured at each treatment session.

### 2.4. Intervention

Treatment was administered by M.A.B.M., a nurse specialist with 30 years of experience in ulcer treatment, using the CAPENERGY Vascular C200 (CE120) tecartherapy device with a C-Boot foot probe in the case of lesions on the sole of the foot, or with capacitive plates in the rest of the body areas.

**Figure 1:**
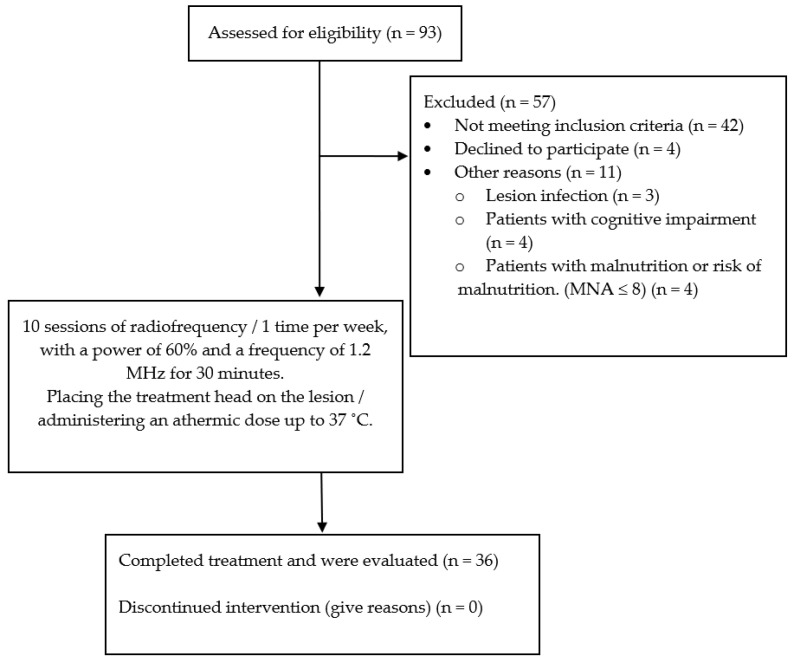
Flow chart of the study.

A total of 10 radiofrequency sessions were applied in the 36 patients with a periodicity of once a week, with a power of 60% and a frequency of 1.2 MHz for 30 min, placing the treatment head on the lesion, and an athermal dose of up to 37 °C was administered (Figure 1).

### 2.5. Statistical Analysis

Statistical analysis was performed using the program R Ver. 3.5.1 (R Foundation for Statistical Computing, Institute for Statistics and Mathematics, Welthandelsplatz 1, 1020 Vienna, Austria). The significance level was set at *p* < 0.05. The distribution of quantitative variables was tested using the Shapiro–Wilk test which evidenced the absence of normality. Qualitative variables were described in absolute values and frequencies and quantitative variables were described using mean and standard deviation.

Given that this is a single-group observational study, the sample size was not calculated a priori, but included patients who attended the ulcer consultation at Hospital Guadarrama (after signing the informed consent and meeting the eligibility criteria) during the period from September 2018 to June 2019. The final power of the study was calculated using the program R Ver. 3.5.1 (R Foundation for Statistical Computing, Institute for Statistics and Mathematics, Welthandelsplatz 1, 1020 Vienna, Austria), applying the Wilcoxon signed-rank test with Bonferroni correction with the PUSH scale scores between the first and last session.

Changes over the sessions in subjective perception and radiofrequency parameters were analyzed and, in the case of radiofrequency parameters, the differences between the right and left legs over the sessions were also analyzed. In both cases, a repeated measures linear mixed model and restricted maximum likelihood (REML) and unstructured correlation (default) structure were used. The subjects were modeled according to random effect and time in the first case, or the group (leg): time interaction in the second as fixed effects, adjusting in the latter case the results with the baseline values. Due to the small sample size, the Kenward–Roger degrees of freedom correction was applied and confidence intervals were calculated via bootstrap. The Nakagawa and Schielzeth R^2^ was calculated for each model as a goodness-of-fit measure. Post hoc matched pair comparisons were applied using the Bonferroni correction.

## 3. Power Analysis

Accepting a risk α of 0.05, the final power of the study was estimated at 100% with a final mean PUSH score of 10.695 at the first session and 4.695 at the ending treatment session.

## 4. Results

The sample was composed of 36 subjects of 63.31 ± 9.99 years, with a majority of women (58.3%) and with risk factors such as diabetes mellitus (66.7%), dyslipidemia (72.2%) or high blood pressure (75.0%) (Table 1).

The recruited patients presented pressure ulcers with an average surface of 25 cm^2^ grade III-IV with an evolution time of approximately 2 months with venous vascular lesions in women and arterial lesions in men.

### 4.1. Comparisons across Sessions

The ANOVA test showed significant differences (*p* < 0.05) throughout the sessions, except in patient satisfaction. It was verified that the presence of systematic differences between practically all of the measurement moments compared with the initial values and specifically between the final values and the initial ones, except in the satisfaction of the patients and the professional and in the frequency in the left leg (*p* < 0.05) (Table 2 and Supplementary Material Table S1).

These differences were translated into a final increase in the values of the radio frequency parameters in both legs and a decrease in the Global Response Assessment score with very high effects on the latter (R2 = 0.874) and being greater than 0.5 in the parameters of the left leg (Supplementary Material Table S2).

The pairwise comparisons showed systematic differences between practically all of the measurement moments, with significant differences being observed in all of the variables between the first and the last session (*p* < 0.05), except in the satisfaction of the patients and the professional (Supplementary Material Table S3).

### 4.2. Comparison of Radiofrequency Parameters between Both Legs

The ANOVA test showed significant differences (*p* < 0.05) between both legs and over time in all parameters except for frequency. The presence of significant differences (*p* < 0.05) was observed over time between legs compared to the initial values in the absorbed energy and in temperature, with higher final values in the absorbed energy in the left leg compared to the right (26.31 ± 3.75 W vs. 17.36 ± 5.66 W) and a moderate effect on both (R2 = 0.471 and 0.492, respectively) (Table 3 and Supplementary Material Table S4).

Pairwise comparisons showed differences in absorbed energy between both legs at the end, with no initial significant differences (*p* = 0.049), while significant differences in temperature occurred in the first treatment sessions (Supplementary Material Table S5).

**Figure 2:**
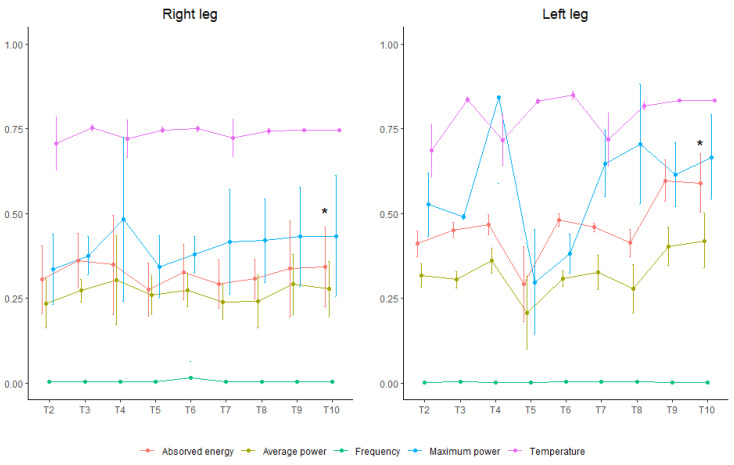
Radiofrequency parameters (standardized adjusted mean and standard deviation error bars). * Radiofrequency parameter significant differences between both legs.

All radiofrequency parameters were found to increase progressively throughout the sessions and more markedly in the left leg, especially with regard to the absorbed energy where the confidence intervals barely overlapped (Figure 2).

### 4.3. Satisfaction of the Patients and the Professional

**Figure 3:**
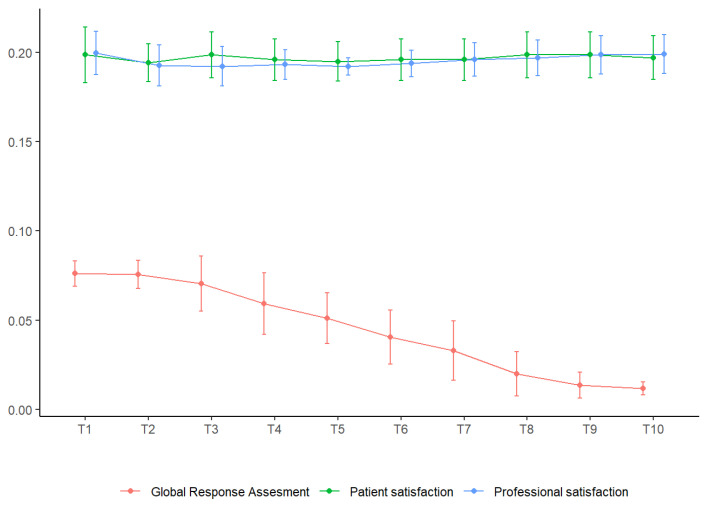
Subjective perception (standardized mean and standard deviation error bars).

A near absence of changes in the satisfaction of both the patients and the professional was observed, while the score in the Global Response Assessment decreased continuously throughout the sessions (Figure 3).

## 5. Discussion

The results of the present study show that an increase in the radiofrequency parameters, including temperature and especially the absorbed energy, especially in the left leg, are indicative of an improvement in the clinical response of the ulcers. Furthermore, a greater exposure to radiofrequency increases the healing power [18,19,20]. It is important to detail that no relevant influences were found with respect to the differences in the results influenced by clinical variables and by sex or any other sociodemographic variables. These results also agree with the previous study [16] in which there was an average increase in temperature by thermography of 1.4 °C, as well as a healing rate percentage of 60% with a progressive reduction in size and exudate of the ulcer measured using the Pressure Ulcer Scale for Healing (PUSH). However, it has not been possible to establish the reason as to why the clinical response of the ulcers in the left leg was better than in the right leg and there are no other studies that have studied the differences between a lower limb and the contralateral limb using tecartherapy. In addition, a review in other fields of electrotherapy such as electrostimulation concluded that there is no consensus on parameters such as frequency, duration and location of treatment [3]. Future studies are therefore needed to study the appropriate dose and whether the differences between one limb and another may be due to tissue factors.

Likewise, for the subjective perception of the results by the patient (GRA), the scores were always higher in patients with dyslipidemia or arterial hypertension compared to those without. In the case of professional satisfaction, patients with renal failure reported higher scores than those without this pathology. We cannot establish hypotheses regarding these results for the moment, on the one hand due to the low sample size of the present study, and on the other hand due to the lack of studies on which to discuss these results. This is because, to the knowledge of the authors of the present study, this is the first time that such comparisons have been made using radiofrequency. This could certainly be a line of research, or at least parameters to be analyzed in future studies.

To our knowledge, this is the first study that uses this type of therapy to address pressure ulcers and assesses parameters of subjective perception of outcomes as well as patient and professional satisfaction. A Cochrane review about electrical stimulation for the treating of pressure ulcers exposes the absence of studies evaluating parameters such as quality of life, depression or perception of treatment effectiveness, despite these being relevant outcomes for patients [3]. In this study, the subjective perception of the results of the treatment measured with the GRA scale worsened, which may be related to the chronic nature of the ulcers and the fact that they did not disappear completely and perhaps did not respond to the expectations of the patients. In this regard, Wood et al. [9], in a study on the collaborative management of pressure ulcers, concluded that patients felt that pressure ulcer care improved by using a collaborative, multidisciplinary approach because they felt more knowledgeable, empowered and more able to improve their pressure ulcer care. This issue was approached from a qualitative perspective by García Sánchez et al. [21], concluding that proximity, trust and effective and bidirectional communication between patients and health professionals are fundamental. Due to a scarcity of studies assessing factors as relevant to the patient as those described above and considering the importance of the patient’s perception in the treatment, future studies concerning these aspects are necessary.

In this sense, Ballestra et al. [22] showed that certain patient expectations, such as the expectation of a tailored treatment with frequent follow-ups, the hope of obtaining the best possible results, realism or resignation regarding the alleviation of the health problem, good dialogue and communication, the need to be seen and confirmed as an individual and the desire to receive an explanation of their disease, could be related to better recovery results.

However, it appears that the induction of different types of expectations (positive or negative) through verbal suggestion does not influence the perception of acute pain perceived during the performance of a technique that may be painful [23].

### Limitations

An important limitation of this study is the small sample size. Additionally important is the absence of a control group or a placebo group to compare with the evolution of the process or with other interventions. The authors also recognize as a limitation the fact that they did not measure the psychological and behavioral factors of the sample analyzed in the study, which is observed as a determinant in different chronic diseases [24,25]. Although there is an inherent bias in the type of research model, this has been minimized by using relevant and reliable human (accredited clinical experience of the researchers) and instrumental resources.

## 6. Conclusions

It was observed that the radiofrequency parameters increased progressively throughout the sessions and more markedly in the left leg, but for the difference between legs it was not possible to establish the reason for this clinical response. This increase was indicative of an improvement in the clinical response to ulcers.

In addition, higher radiofrequency exposure increases healing capacity.

However, the subjective perception of treatment outcomes worsened, which may be related to the chronic nature of the ulcers, leading to patients’ expectations not being met.

## 7. Key Points

In summary, considering the great clinical potential of radiofrequency, we can expect an increase in new techniques for tissue regeneration and wound healing in the near future.

The increased power to accelerate the wound healing process can be explained by the anti-inflammatory effect caused by the changes that occur in the perilesional skin, and the improvement in microcirculation contributes to the increase in the reactivity of the different layers of the skin.

The influence of the magnetic field on the microcirculatory system can be used to explain the often-cited fact that magnetic fields have anti-oedematous, analgesic and anti-inflammatory effects, which is one of the reasons for their wide application in the field of injury treatment.

The subjective perception of treatment success may be influenced by the chronic nature of the ulcers, which leads to patients’ expectations not being met.

## Figures and Tables

**Table 1:** Clinical and demographic characteristics of the participants.

n		36
Age		63.31 ± 9.9
Weight		72.25 ± 8.8
Height		162.33 ± 3.8
Gender, n(%)	Female	21 (58)
	Male	15 (41)
Smoking, n(%)	No	36 (100)
Embolism, n(%)	No	36 (100)
Diabetes mellitus, n(%)	No	12 (33)
	Yes	24 (66)
Dyslipidemia, n(%)	No	10 (28)
	Yes	26 (72)
Arterial hypertension, n(%)	No	9 (25)
	Yes	27 (75)
Kidney failure, n(%)	No	34 (94)
	Yes	2 (6)
Parkinson’s, n(%)	No	36 (100)
Alzheimer’s, n(%)	No	36 (100)
Dementia, n(%)	No	36 (100)
Respiratory pathology, n(%)	No	36 (100)
Cardiac pathology, n(%)	No	36 (100)
Musculoskeletal pathology, n(%)	No	36 (100)
Other pathologies, n(%)	No	36 (100)
Nutritional supplements, n(%)	No	36 (100)
Muscle relaxants, n(%)	No	36 (100)
Anxiolytics, n(%)	No	35 (97)
	Yes	1 (3)
Antiepileptics, n(%)	No	34 (94)
	Yes	2 (6)
Analgesics, n(%)	No	30 (83.3)
	Yes	6 (17)
Opioids, n(%)	No	36 (100)
Statins, n(%)	No	36 (100)
Antiaggregants, n(%)	No	36 (100)
Fever, n(%)	No	36 (100.0)
Pain, n(%)	No	30 (83.3)
	Yes	6 (16.7)
Traumatism, n(%)	No	36 (100.0)

Data expressed as mean ± standard deviation or with absolute and relative values (%).

**Table 2:** Subjective perception and radiofrequency parameters: model results.

	Global Response Assessment	Patients’ Satisfaction	Professional Satisfaction	Right Frequency (Hz)	Right Maximum Power (Watts)	Right Average Power (Watts)	Right Absorbed Energy (Volt-Ampere)	Right Temperature (Celsius)	Left Frequency (Hz)	Left Maximum Power (Watts)	Left Average Power (Watts)	Left Absorbed Energy (Volt-Ampere)	Left Temperature (Celsius)
Nakagawa and Schielzeth R^2^	0.874	0.397	0.437	0.081	0.435	0.276	0.401	0.344	0.687	0.676	0.564	0.708	0.729
T10-T1 difference (95%CI)	−2.861 (−2.98, −2.742)	−0.071 (−0.333, 0.19)	−0.024 (−0.224, 0.177)	14.917 (12.698, 17.136)	13.612 (10.063, 17.162)	8.596 (6.009, 11.183)	6.03 (5.142, 6.918)	−33.15 (−34.464, −31.837)	−0.03 (−0.076, 0.015)	21.956 (15.106, 28.805)	21.166 (17.509, 24.822)	25.333 (24.726, 25.939)	1.833 (0.792, 2.873)
Omnibus ANOVA: time	F(9, 315) = 247.659, *p* = <0.001	F(9, 369) = 1.261, *p* = 0.257	F(9, 369) = 6.346, *p* = <0.001	F(9, 369) = 4.126, *p* = <0.001	F(9, 369) = 11.563, *p* = <0.001	F(9, 369) = 6.852, *p* = <0.001	F(9, 369) = 6.919, *p* = <0.001	F(9, 369) = 16.484, *p* = <0.001	F(9, 81) = 15.793, *p* = <0.001	F(9, 81) = 20.876, *p* = <0.001	F(9, 81) = 13.503, *p* = <0.001	F(9, 81) = 26.622, *p* = <0.001	F(9, 81) = 25.628, *p* = <0.001

F: F statistic (degrees of freedom); 95%CI: 95% confidence interval. Significant if *p* < 0.05 (shown in red).

**Table 3:** Radiofrequency parameters between both legs: model results.

	Frequency (Hz)	Maximum Power (Watts)	Average Power (Watts)	Absorbed Energy (Volt-Ampere)	Temperature (Celsius)
Nakagawa and Schielzeth R^2^	0.058	0.522	0.301	0.471	0.492
Omnibus ANOVA: group:time	F(8, 412.104) = 0.368, *p* = 0.937	F(8, 409.049) = 8.882, *p* = <0.001	F(8, 411.292) = 5.702, *p* = <0.001	F(8, 409.197) = 8.484, *p* = <0.001	F(8, 409.175) = 12.53, *p* = <0.001
T10 between groups’ adjusted difference (95%CI)	0.2 (−1.61, 2.01)	−0.3 (−1.421, 0.821)	−0.02 (−0.065, 0.025)	4.3 (2.304, 6.296)	<0.001 (< 0.001, <0.001)

F: F statistic (degrees of freedom); 95%CI: 95% confidence interval; 95%CI: 95% confidence interval. Significant if p < 0.05 (shown in red).

## Data Availability

The data presented in this study are available upon request from the corresponding authors. The data are not publicly available due to ethical restrictions.

## Supplementary Materials

The following supporting information can be downloaded at https://www.mdpi.com/article/10.3390/medicina59030516/s1: Table S1: Subjective perception and radiofrequency parameters model coefficient terms results; Table S2: Subjective perception and radiofrequency parameters outcomes; Table S3: Subjective perception and radiofrequency parameters overall pairwise model coefficient terms results; Table S4: Radiofrequency parameters between both legs model coefficient terms results; Table S5: Radiofrequency parameters between both legs model pairwise results.
