# Supplementary material for: Presence of Differences in the Radiofrequency Parameters Applied to Complex Pressure Ulcers: A Secondary Analysis
Source: Medicina (Kaunas). 2023 Mar 7;59(3):516. doi: 10.3390/medicina59030516 (PMC10059019; doi:10.3390/medicina59030516)
Supplement: Supplementary file 1 [file medicina-59-00516-s001.zip › medicina-2181607-supplementary.pdf]

Supplementary Material

Supplementary material. Table 1: Subjective perception and radiofrequency parameters model coefficient terms results.

	Global Response Assesment	Patients satisfaction	Professional satisfaction	Right frequency (Hz)	Right maximum power (Watts)	Right average power (Watts)	Right absorbed energy (Volt-ampere)	Right temperatura (Celsius)	Left frequency (Hz)	Left maximum power (Watts)	Left average power (Watts)	Left absorbed energy (Volt-ampere)	Left temperatura (Celsius)
(Intercept)	3.889 (SE=0.093) 95%CI(3.686, 4.101), t=41.929, p=<0.001	9.333 (SE=0.085) 95%CI(9.174, 9.52), t=110.146, p=<0.001	9.381 (SE=0.068) 95%CI(9.201, 9.517), t=138.772, p=<0.001	2.448 (SE=0.208) 95%CI(1.95, 2.944), t=11.773, p=<0.001	11.081 (SE=0.986) 95%CI(9.239, 12.845), t=11.241, p=<0.001	8.05 (SE=0.503) 95%CI(7.137, 8.86), t=15.997, p=<0.001	8.214 (SE=0.622) 95%CI(7.254, 9.448), t=13.199, p=<0.001	33.762 (SE=0.306) 95%CI(33.048, 34.529), t=110.276, p=<0.001	1.06 (SE=0.024) 95%CI(1.022, 1.116), t=44.749, p=<0.001	11.9 (SE=1.783) 95%CI(8.43, 15.497), t=6.675, p=<0.001	8.39 (SE=0.759) 95%CI(6.823, 9.818), t=11.047, p=<0.001	11.4 (SE=0.73) 95%CI(9.954, 12.818), t=15.615, p=<0.001	34.9 (SE=0.598) 95%CI(33.656, 36.039), t=58.356, p=<0.001
T2	-0.028 (SE=0.1) 95%CI(-0.194, 0.189), t=-0.277, p=0.782	-0.19 (SE=0.094) 95%CI(-0.372, -0.022), t=-2.03, p=0.043	-0.31 (SE=0.075) 95%CI(-0.478, -0.131), t=-4.145, p=<0.001	-1.286 (SE=0.294) 95%CI(-2.034, -0.585), t=-4.373, p=<0.001	2.064 (SE=1.13) 95%CI(-0.129, 4.309), t=1.826, p=0.069	0.105 (SE=0.641) 95%CI(-1.098, 1.26), t=0.163, p=0.87	0.186 (SE=0.714) 95%CI(-1.085, 2.001), t=0.26, p=0.795	1.31 (SE=0.4) 95%CI(0.362, 2.199), t=3.271, p=0.001	-0.02 (SE=0.025) 95%CI(-0.072, 0.025), t=-0.792, p=0.431	7.6 (SE=2.316) 95%CI(3.465, 11.68), t=3.282, p=0.002	1.91 (SE=1.04) 95%CI(-0.47, 3.684), t=1.837, p=0.07	-1.6 (SE=1.032) 95%CI(-3.677, 0.578), t=-1.55, p=0.125	-4.2 (SE=0.725) 95%CI(-5.653, -2.985), t=-5.793, p=<0.001
T3	-0.25 (SE=0.1) 95%CI(-0.453, -0.062), t=-2.493, p=0.013	0 (SE=0.094) 95%CI(-0.188, 0.204), t=0, p=>0.999	-0.333 (SE=0.075) 95%CI(-0.496, -0.177), t=-4.464, p=<0.001	-1.271 (SE=0.294) 95%CI(-1.951, -0.635), t=-4.324, p=<0.001	4.014 (SE=1.13) 95%CI(1.728, 6.187), t=3.551, p=<0.001	1.902 (SE=0.641) 95%CI(0.619, 3.351), t=2.969, p=0.003	2.857 (SE=0.714) 95%CI(1.554, 4.335), t=4.003, p=<0.001	3.5 (SE=0.4) 95%CI(2.665, 4.366), t=8.741, p=<0.001	0.14 (SE=0.025) 95%CI(0.09, 0.196), t=5.546, p=<0.001	6.1 (SE=2.316) 95%CI(1.744, 11.061), t=2.634, p=0.01	1.41 (SE=1.04) 95%CI(-0.479, 3.991), t=1.356, p=0.179	0.1 (SE=1.032) 95%CI(-1.747, 2.207), t=0.097, p=0.923	2.2 (SE=0.725) 95%CI(0.779, 3.814), t=3.034, p=0.003
T4	-0.75 (SE=0.1) 95%CI(-0.961, -0.543), t=-7.478, p=<0.001	-0.119 (SE=0.094) 95%CI(-0.313, 0.1), t=-1.269, p=0.205	-0.286 (SE=0.075) 95%CI(-0.47, -0.122), t=-3.826, p=<0.001	-1.31 (SE=0.294) 95%CI(-1.911, -0.587), t=-4.454, p=<0.001	9.133 (SE=1.13) 95%CI(6.907, 11.411), t=8.08, p=<0.001	3.379 (SE=0.641) 95%CI(2.361, 4.853), t=5.273, p=<0.001	2.279 (SE=0.714) 95%CI(0.928, 3.753), t=3.193, p=0.002	2.024 (SE=0.4) 95%CI(1.107, 2.836), t=5.055, p=<0.001	0 (SE=0.025) 95%CI(-0.06, 0.06), t=0, p=>0.999	21.2 (SE=2.316) 95%CI(16.2, 24.986), t=9.156, p=<0.001	3.81 (SE=1.04) 95%CI(1.831, 6.278), t=3.664, p=<0.001	0.8 (SE=1.032) 95%CI(-1.158, 3.466), t=0.775, p=0.441	-2.9 (SE=0.725) 95%CI(-4.648, -1.355), t=-4, p=<0.001
T5	-1.111 (SE=0.1) 95%CI(-1.322, -0.938), t=-11.079, p=<0.001	-0.167 (SE=0.094) 95%CI(-0.389, -0.032), t=-1.777, p=0.076	-0.333 (SE=0.075) 95%CI(-0.479, -0.17), t=-4.464, p=<0.001	-1.281 (SE=0.294) 95%CI(-2.003, -0.74), t=-4.357, p=<0.001	2.443 (SE=1.13) 95%CI(0.292, 4.673), t=2.161, p=0.031	1.26 (SE=0.641) 95%CI(0.035, 2.597), t=1.966, p=0.05	-1.252 (SE=0.714) 95%CI(-2.752, 0.341), t=-1.755, p=0.08	3.262 (SE=0.4) 95%CI(2.402, 4.079), t=8.147, p=<0.001	0 (SE=0.025) 95%CI(-0.063, 0.052), t=0, p=>0.999	-2.2 (SE=2.316) 95%CI(-6.875, 2.953), t=-0.95, p=0.345	-2.79 (SE=1.04) 95%CI(-4.893, -1.109), t=-2.683, p=0.009	-6.7 (SE=1.032) 95%CI(-8.859, -4.345), t=-6.489, p=<0.001	2 (SE=0.725) 95%CI(0.349, 3.492), t=2.759, p=0.007
T6	-1.583 (SE=0.1) 95%CI(-1.784, -1.379), t=-15.787, p=<0.001	-0.119 (SE=0.094) 95%CI(-0.31, 0.062), t=-1.269, p=0.205	-0.262 (SE=0.075) 95%CI(-0.444, -0.119), t=-3.507, p=0.001	-0.771 (SE=0.294) 95%CI(-1.522, -0.137), t=-2.624, p=0.009	4.157 (SE=1.13) 95%CI(2.497, 6.238), t=3.677, p=<0.001	1.974 (SE=0.641) 95%CI(1.011, 3.53), t=3.08, p=0.002	1.238 (SE=0.714) 95%CI(-0.216, 2.949), t=1.735, p=0.084	3.405 (SE=0.4) 95%CI(2.496, 4.218), t=8.504, p=<0.001	0.14 (SE=0.025) 95%CI(0.079, 0.19), t=5.546, p=<0.001	1.4 (SE=2.316) 95%CI(-2.831, 5.088), t=0.605, p=0.547	1.51 (SE=1.04) 95%CI(-1.14, 3.586), t=1.452, p=0.15	1.4 (SE=1.032) 95%CI(-0.246, 3.379), t=1.356, p=0.179	2.8 (SE=0.725) 95%CI(1.178, 4.313), t=3.862, p=<0.001
T7	-1.917 (SE=0.1) 95%CI(-2.102, -1.7), t=-19.111, p=<0.001	-0.119 (SE=0.094) 95%CI(-0.334, 0.044), t=-1.269, p=0.205	-0.167 (SE=0.075) 95%CI(-0.303, -0.018), t=-2.232, p=0.026	-1.257 (SE=0.294) 95%CI(-1.884, -0.573), t=-4.276, p=<0.001	5.967 (SE=1.13) 95%CI(3.623, 8.631), t=5.278, p=<0.001	0.331 (SE=0.641) 95%CI(-0.938, 1.7), t=0.516, p=0.606	-0.483 (SE=0.714) 95%CI(-2.377, 0.825), t=-0.677, p=0.499	2.071 (SE=0.4) 95%CI(1.29, 2.86), t=5.174, p=<0.001	0.14 (SE=0.025) 95%CI(0.072, 0.197), t=5.546, p=<0.001	12.8 (SE=2.316) 95%CI(8.367, 17.586), t=5.528, p=<0.001	2.31 (SE=1.04) 95%CI(0.215, 4.379), t=2.221, p=0.029	0.5 (SE=1.032) 95%CI(-1.232, 2.704), t=0.484, p=0.629	-2.8 (SE=0.725) 95%CI(-4.33, -0.863), t=-3.862, p=<0.001
T8	-2.5 (SE=0.1) 95%CI(-2.724, -2.306), t=-24.927, p=<0.001	0 (SE=0.094) 95%CI(-0.194, 0.176), t=0, p=>0.999	-0.119 (SE=0.075) 95%CI(-0.265, 0.071), t=-1.594, p=0.112	-1.281 (SE=0.294) 95%CI(-1.922, -0.561), t=-4.357, p=<0.001	6.157 (SE=1.13) 95%CI(4.136, 8.058), t=5.447, p=<0.001	0.429 (SE=0.641) 95%CI(-0.517, 1.99), t=0.669, p=0.504	0.262 (SE=0.714) 95%CI(-1.303, 1.849), t=0.367, p=0.714	3.071 (SE=0.4) 95%CI(2.114, 3.999), t=7.671, p=<0.001	0.11 (SE=0.025) 95%CI(0.044, 0.179), t=4.357, p=<0.001	15.3 (SE=2.316) 95%CI(11.535, 20.914), t=6.608, p=<0.001	0.21 (SE=1.04) 95%CI(-1.643, 2.965), t=0.202, p=0.84	-1.5 (SE=1.032) 95%CI(-3.163, 0.53), t=-1.453, p=0.15	1.4 (SE=0.725) 95%CI(-0.022, 2.977), t=1.931, p=0.057
T9	-2.778 (SE=0.1) 95%CI(-2.984, -2.576), t=-27.696, p=<0.001	0 (SE=0.094) 95%CI(-0.183, 0.16), t=0, p=>0.999	-0.048 (SE=0.075) 95%CI(-0.186, 0.094), t=-0.638, p=0.524	-1.31 (SE=0.294) 95%CI(-1.967, -0.658), t=-4.454, p=<0.001	6.705 (SE=1.13) 95%CI(4.863, 8.858), t=5.931, p=<0.001	2.783 (SE=0.641) 95%CI(1.482, 4.187), t=4.344, p=<0.001	1.7 (SE=0.714) 95%CI(0.184, 3.531), t=2.382, p=0.018	3.238 (SE=0.4) 95%CI(2.466, 4.13), t=8.087, p=<0.001	0 (SE=0.025) 95%CI(-0.057, 0.055), t=0, p=>0.999	11.4 (SE=2.316) 95%CI(7.999, 16.068), t=4.923, p=<0.001	5.61 (SE=1.04) 95%CI(3.321, 7.882), t=5.395, p=<0.001	6.4 (SE=1.032) 95%CI(4.46, 8.726), t=6.199, p=<0.001	2.1 (SE=0.725) 95%CI(0.439, 3.734), t=2.896, p=0.005
T10	-2.861 (SE=0.1) 95%CI(-3.08, -2.667), t=-28.527, p=<0.001	-0.071 (SE=0.094) 95%CI(-0.3, 0.103), t=-0.761, p=0.447	-0.024 (SE=0.075) 95%CI(-0.188, 0.138), t=-0.319, p=0.75	-1.314 (SE=0.294) 95%CI(-1.94, -0.932), t=-4.47, p=<0.001	6.752 (SE=1.13) 95%CI(4.643, 8.932), t=5.973, p=<0.001	2.117 (SE=0.641) 95%CI(0.94, 3.572), t=3.303, p=0.001	1.952 (SE=0.714) 95%CI(0.453, 3.179), t=2.735, p=0.007	3.238 (SE=0.4) 95%CI(2.196, 4.177), t=8.087, p=<0.001	0 (SE=0.025) 95%CI(-0.065, 0.058), t=0, p=>0.999	13.6 (SE=2.316) 95%CI(8.811, 18.472), t=5.873, p=<0.001	6.31 (SE=1.04) 95%CI(3.658, 8.961), t=6.068, p=<0.001	6.1 (SE=1.032) 95%CI(4.143, 8.421), t=5.908, p=<0.001	2.1 (SE=0.725) 95%CI(0.449, 3.469), t=2.896, p=0.005

(SE): model coefficient (standard error); 95%CI: 95% confidence interval; t: Significant if p<0.05 (shown in red).

Supplementary material. Table 2: Subjective perception and radiofrequency parameters outcomes.

	**T1**	**T2**	**T3**	**T4**	**T5**	**T6**	**T7**	**T8**	**T9**	**T10**
Global Response Assesment	3.89±0.32	3.86±0.35	3.64±0.68	3.14±0.76	2.78±0.64	2.31±0.67	1.97±0.74	1.39±0.55	1.11±0.32	1.03±0.17
Patients satisfaction	9.33±0.69	9.14±0.47	9.33±0.57	9.21±0.52	9.17±0.49	9.21±0.52	9.21±0.52	9.33±0.57	9.33±0.57	9.26±0.54
Professional satisfaction	9.38±0.54	9.07±0.51	9.05±0.49	9.10±0.37	9.05±0.22	9.12±0.33	9.21±0.42	9.26±0.45	9.33±0.48	9.36±0.48
Right frequency (Hz)	2.45±3.55	1.15±0.08	1.16±0.07	1.12±0.09	1.15±0.07	1.66±2.34	1.18±0.04	1.15±0.07	1.12±0.09	1.12±0.10
Right maximum power (Watts)	11.08±4.64	17.02±4.97	18.97±2.70	24.09±11.60	17.40±4.39	19.12±2.56	20.93±7.39	21.12±5.89	21.66±7.03	21.71±8.54
Right average power (Watts)	8.05±3.97	12.23±3.48	14.03±1.62	15.51±6.28	13.39±2.73	14.10±2.38	12.46±2.51	12.56±3.74	14.91±4.22	14.24±3.94
Right absorved energy (Volt-ampere)	8.21±4.11	15.60±4.82	18.27±3.82	17.69±7.01	14.16±3.76	16.65±3.90	14.93±3.44	15.67±2.81	17.11±6.77	17.36±5.66
Right temperatura (Celsius)	34.90±1.45	30.43±3.33	36.83±0.31	31.73±3.23	36.63±0.32	37.43±0.49	31.83±3.38	36.03±0.48	36.73±0.01	36.73±0.01
Left frequency (Hz)	1.06±0.10	1.03±0.08	1.19±0.00	1.05±0.10	1.05±0.10	1.19±0.00	1.19±0.00	1.16±0.05	1.05±0.10	1.05±0.10
Left maximum power (Watts)	11.90±1.66	23.56±3.97	22.06±0.29	37.16±10.90	13.76±6.63	17.36±2.45	28.76±4.20	31.26±7.53	27.36±4.01	29.56±5.38
Left average power (Watts)	8.39±2.66	14.60±1.49	14.10±1.03	16.50±1.61	9.90±4.69	14.20±1.02	15.00±2.20	12.90±3.07	18.30±2.39	19.00±3.48
Left absorved energy (Volt-ampere)	11.40±0.84	18.61±1.66	20.31±0.99	21.01±1.27	13.51±4.75	21.61±0.79	20.71±0.53	18.71±1.75	26.61±2.65	26.31±3.75
Left temperatura (Celsius)	34.90±1.45	30.43±3.33	36.83±0.31	31.73±3.23	36.63±0.32	37.43±0.49	31.83±3.38	36.03±0.48	36.73±0.01	36.73±0.01

Data expressed as mean±standard deviation.

Supplementary material. Table 3: Subjective perception and radiofrequency parameters overall pairwise model coefficient terms results.

	Global Response Assesment	Patients satisfaction	Professional satisfaction	Right frequency (Hz)	Right maximum power (Watts)	Right average power (Watts)	Right absorved energy (Volt-ampere)	Right temperatura (Celsius)	Left frequency (Hz)	Left maximum power (Watts)	Left average power (Watts)	Left absorved energy (Volt-ampere)	Left temperatura (Celsius)
T1-T10	2.861 (SE=0.1) 95%CI(2.664, 3.058), t=28.527, p=<0.001	0.071 (SE=0.094) 95%CI(-0.113, 0.256), t=0.761, p=0.447	0.024 (SE=0.075) 95%CI(-0.123, 0.171), t=0.319, p=0.75	1.314 (SE=0.294) 95%CI(0.736, 1.892), t=4.47, p=<0.001	-6.752 (SE=1.13) 95%CI(-8.975, -4.529), t=-5.973, p=<0.001	-2.117 (SE=0.641) 95%CI(-3.377, -0.857), t=-3.303, p=0.001	-1.952 (SE=0.714) 95%CI(-3.356, -0.549), t=-2.735, p=0.007	-3.238 (SE=0.4) 95%CI(-4.025, -2.451), t=-8.087, p=<0.001	0 (SE=0.025) 95%CI(-0.05, 0.05), t=0, p=>0.999	-13.6 (SE=2.316) 95%CI(-18.207, -8.993), t=-5.873, p=<0.001	-6.31 (SE=1.04) 95%CI(-8.379, -4.241), t=-6.068, p=<0.001	-6.1 (SE=1.032) 95%CI(-8.154, -4.046), t=-5.908, p=<0.001	-2.1 (SE=0.725) 95%CI(-3.543, -0.657), t=-2.896, p=0.005
T1-T2	0.028 (SE=0.1) 95%CI(-0.17, 0.225), t=0.277, p=0.782	0.19 (SE=0.094) 95%CI(0.006, 0.375), t=2.03, p=0.043	0.31 (SE=0.075) 95%CI(0.163, 0.456), t=4.145, p=<0.001	1.286 (SE=0.294) 95%CI(0.708, 1.864), t=4.373, p=<0.001	-2.064 (SE=1.13) 95%CI(-4.287, 0.159), t=-1.826, p=0.069	-0.105 (SE=0.641) 95%CI(-1.365, 1.155), t=-0.163, p=0.87	-0.186 (SE=0.714) 95%CI(-1.589, 1.218), t=-0.26, p=0.795	-1.31 (SE=0.4) 95%CI(-2.097, -0.522), t=-3.271, p=0.001	0.02 (SE=0.025) 95%CI(-0.03, 0.07), t=0.792, p=0.431	-7.6 (SE=2.316) 95%CI(-12.207, -2.993), t=-3.282, p=0.002	-1.91 (SE=1.04) 95%CI(-3.979, 0.159), t=-1.837, p=0.07	1.6 (SE=1.032) 95%CI(-0.454, 3.654), t=1.55, p=0.125	4.2 (SE=0.725) 95%CI(2.757, 5.643), t=5.793, p=<0.001
T1-T3	0.25 (SE=0.1) 95%CI(0.053, 0.447), t=2.493, p=0.013	0 (SE=0.094) 95%CI(-0.184, 0.184), t=0, p=>0.999	0.333 (SE=0.075) 95%CI(0.186, 0.48), t=4.464, p=<0.001	1.271 (SE=0.294) 95%CI(0.693, 1.85), t=4.324, p=<0.001	-4.014 (SE=1.13) 95%CI(-6.237, -1.791), t=-3.551, p=<0.001	-1.902 (SE=0.641) 95%CI(-3.162, -0.642), t=-2.969, p=0.003	-2.857 (SE=0.714) 95%CI(-4.261, -1.454), t=-4.003, p=<0.001	-3.5 (SE=0.4) 95%CI(-4.287, -2.713), t=-8.741, p=<0.001	-0.14 (SE=0.025) 95%CI(-0.19, -0.09), t=-5.546, p=<0.001	-6.1 (SE=2.316) 95%CI(-10.707, -1.493), t=-2.634, p=0.01	-1.41 (SE=1.04) 95%CI(-3.479, 0.659), t=-1.356, p=0.179	-0.1 (SE=1.032) 95%CI(-2.154, 1.954), t=-0.097, p=0.923	-2.2 (SE=0.725) 95%CI(-3.643, -0.757), t=-3.034, p=0.003
T1-T4	0.75 (SE=0.1) 95%CI(0.553, 0.947), t=7.478, p=<0.001	0.119 (SE=0.094) 95%CI(-0.065, 0.304), t=1.269, p=0.205	0.286 (SE=0.075) 95%CI(0.139, 0.433), t=3.826, p=<0.001	1.31 (SE=0.294) 95%CI(0.731, 1.888), t=4.454, p=<0.001	-9.133 (SE=1.13) 95%CI(-11.356, -6.91), t=-8.08, p=<0.001	-3.379 (SE=0.641) 95%CI(-4.639, -2.119), t=-5.273, p=<0.001	-2.279 (SE=0.714) 95%CI(-3.682, -0.875), t=-3.193, p=0.002	-2.024 (SE=0.4) 95%CI(-2.811, -1.236), t=-5.055, p=<0.001	0 (SE=0.025) 95%CI(-0.05, 0.05), t=0, p=>0.999	-21.2 (SE=2.316) 95%CI(-25.807, -16.593), t=-9.156, p=<0.001	-3.81 (SE=1.04) 95%CI(-5.879, -1.741), t=-3.664, p=<0.001	-0.8 (SE=1.032) 95%CI(-2.854, 1.254), t=-0.775, p=0.441	2.9 (SE=0.725) 95%CI(1.457, 4.343), t=4, p=<0.001
T1-T5	1.111 (SE=0.1) 95%CI(0.914, 1.308), t=11.079, p=<0.001	0.167 (SE=0.094) 95%CI(-0.018, 0.351), t=1.777, p=0.076	0.333 (SE=0.075) 95%CI(0.186, 0.48), t=4.464, p=<0.001	1.281 (SE=0.294) 95%CI(0.703, 1.859), t=4.357, p=<0.001	-2.443 (SE=1.13) 95%CI(-4.666, -0.22), t=-2.161, p=0.031	-1.26 (SE=0.641) 95%CI(-2.52, 0), t=-1.966, p=0.05	1.252 (SE=0.714) 95%CI(-0.151, 2.656), t=1.755, p=0.08	-3.262 (SE=0.4) 95%CI(-4.049, -2.475), t=-8.147, p=<0.001	0 (SE=0.025) 95%CI(-0.05, 0.05), t=0, p=>0.999	2.2 (SE=2.316) 95%CI(-2.407, 6.807), t=0.95, p=0.345	2.79 (SE=1.04) 95%CI(0.721, 4.859), t=2.683, p=0.009	6.7 (SE=1.032) 95%CI(4.646, 8.754), t=6.489, p=<0.001	-2 (SE=0.725) 95%CI(-3.443, -0.557), t=-2.759, p=0.007
T1-T6	1.583 (SE=0.1) 95%CI(1.386, 1.781), t=15.787, p=<0.001	0.119 (SE=0.094) 95%CI(-0.065, 0.304), t=1.269, p=0.205	0.262 (SE=0.075) 95%CI(0.115, 0.409), t=3.507, p=0.001	0.771 (SE=0.294) 95%CI(0.193, 1.35), t=2.624, p=0.009	-4.157 (SE=1.13) 95%CI(-6.38, -1.934), t=-3.677, p=<0.001	-1.974 (SE=0.641) 95%CI(-3.234, -0.714), t=-3.08, p=0.002	-1.238 (SE=0.714) 95%CI(-2.642, 0.165), t=-1.735, p=0.084	-3.405 (SE=0.4) 95%CI(-4.192, -2.617), t=-8.504, p=<0.001	-0.14 (SE=0.025) 95%CI(-0.19, -0.09), t=-5.546, p=<0.001	-1.4 (SE=2.316) 95%CI(-6.007, 3.207), t=-0.605, p=0.547	-1.51 (SE=1.04) 95%CI(-3.579, 0.559), t=-1.452, p=0.15	-1.4 (SE=1.032) 95%CI(-3.454, 0.654), t=-1.356, p=0.179	-2.8 (SE=0.725) 95%CI(-4.243, -1.357), t=-3.862, p=<0.001
T1-T7	1.917 (SE=0.1) 95%CI(1.719, 2.114), t=19.111, p=<0.001	0.119 (SE=0.094) 95%CI(-0.065, 0.304), t=1.269, p=0.205	0.167 (SE=0.075) 95%CI(0.02, 0.314), t=2.232, p=0.026	1.257 (SE=0.294) 95%CI(0.679, 1.835), t=4.276, p=<0.001	-5.967 (SE=1.13) 95%CI(-8.19, -3.744), t=-5.278, p=<0.001	-0.331 (SE=0.641) 95%CI(-1.591, 0.929), t=-0.516, p=0.606	0.483 (SE=0.714) 95%CI(-0.92, 1.887), t=0.677, p=0.499	-2.071 (SE=0.4) 95%CI(-2.859, -1.284), t=-5.174, p=<0.001	-0.14 (SE=0.025) 95%CI(-0.19, -0.09), t=-5.546, p=<0.001	-12.8 (SE=2.316) 95%CI(-17.407, -8.193), t=-5.528, p=<0.001	-2.31 (SE=1.04) 95%CI(-4.379, -0.241), t=-2.221, p=0.029	-0.5 (SE=1.032) 95%CI(-2.554, 1.554), t=-0.484, p=0.629	2.8 (SE=0.725) 95%CI(1.357, 4.243), t=3.862, p=<0.001
T1-T8	2.5 (SE=0.1) 95%CI(2.303, 2.697), t=24.927, p=<0.001	0 (SE=0.094) 95%CI(-0.184, 0.184), t=0, p=>0.999	0.119 (SE=0.075) 95%CI(-0.028, 0.266), t=1.594, p=0.112	1.281 (SE=0.294) 95%CI(0.703, 1.859), t=4.357, p=<0.001	-6.157 (SE=1.13) 95%CI(-8.38, -3.934), t=-5.447, p=<0.001	-0.429 (SE=0.641) 95%CI(-1.689, 0.831), t=-0.669, p=0.504	-0.262 (SE=0.714) 95%CI(-1.665, 1.142), t=-0.367, p=0.714	-3.071 (SE=0.4) 95%CI(-3.859, -2.284), t=-7.671, p=<0.001	-0.11 (SE=0.025) 95%CI(-0.16, -0.06), t=-4.357, p=<0.001	-15.3 (SE=2.316) 95%CI(-19.907, -10.693), t=-6.608, p=<0.001	-0.21 (SE=1.04) 95%CI(-2.279, 1.859), t=-0.202, p=0.84	1.5 (SE=1.032) 95%CI(-0.554, 3.554), t=1.453, p=0.15	-1.4 (SE=0.725) 95%CI(-2.843, 0.043), t=-1.931, p=0.057
T1-T9	2.778 (SE=0.1) 95%CI(2.58,	0 (SE=0.094) 95%CI(-	0.048 (SE=0.075) 95%CI(-	1.31 (SE=0.294) 95%CI(0.731,	-6.705 (SE=1.13) 95%CI(-	-2.783 (SE=0.641) 95%CI(-	-1.7 (SE=0.714) 95%CI(-	-3.238 (SE=0.4) 95%CI(-	0 (SE=0.025) 95%CI(-0.05,	-11.4 (SE=2.316) 95%CI(-	-5.61 (SE=1.04) 95%CI(-	-6.4 (SE=1.032) 95%CI(-	-2.1 (SE=0.725) 95%CI(-3.543,

	2.975), t=27.696, p=<0.001	0.184, 0.184), t=0, p=>0.999	0.099, 0.194), t=0.638, p=0.524	1.888), t=4.454, p=<0.001	8.928, -4.482), t=-5.931, p=<0.001	4.043, -1.523), t=-4.344, p=<0.001	3.103, -0.297), t=-2.382, p=0.018	4.025, -2.451), t=-8.087, p=<0.001	0.05), t=0, p=>0.999	16.007, -6.793), t=-4.923, p=<0.001	7.679, -3.541), t=-5.395, p=<0.001	8.454, -4.346), t=-6.199, p=<0.001	-0.657), t=-2.896, p=0.005
T2-T10	2.833 (SE=0.1) 95%CI(2.636, 3.031), t=28.25, p=<0.001	-0.119 (SE=0.094) 95%CI(-0.304, 0.065), t=-1.269, p=0.205	-0.286 (SE=0.075) 95%CI(-0.433, -0.139), t=-3.826, p=<0.001	0.029 (SE=0.294) 95%CI(-0.55, 0.607), t=0.097, p=0.923	-4.688 (SE=1.13) 95%CI(-6.911, -2.465), t=-4.147, p=<0.001	-2.012 (SE=0.641) 95%CI(-3.272, -0.752), t=-3.14, p=0.002	-1.767 (SE=0.714) 95%CI(-3.17, -0.363), t=-2.475, p=0.014	-1.929 (SE=0.4) 95%CI(-2.716, -1.141), t=-4.817, p=<0.001	-0.02 (SE=0.025) 95%CI(-0.07, 0.03), t=-0.792, p=0.431	-6 (SE=2.316) 95%CI(-10.607, -1.393), t=-2.591, p=0.011	-4.4 (SE=1.04) 95%CI(-6.469, -2.331), t=-4.231, p=<0.001	-7.7 (SE=1.032) 95%CI(-9.754, -5.646), t=-7.458, p=<0.001	-6.3 (SE=0.725) 95%CI(-7.743, -4.857), t=-8.689, p=<0.001
T2-T3	0.222 (SE=0.1) 95%CI(0.025, 0.42), t=2.216, p=0.027	-0.19 (SE=0.094) 95%CI(-0.375, -0.006), t=-2.03, p=0.043	0.024 (SE=0.075) 95%CI(-0.123, 0.171), t=0.319, p=0.75	-0.014 (SE=0.294) 95%CI(-0.592, 0.564), t=-0.049, p=0.961	-1.95 (SE=1.13) 95%CI(-4.173, 0.273), t=-1.725, p=0.085	-1.798 (SE=0.641) 95%CI(-3.058, -0.538), t=-2.805, p=0.005	-2.671 (SE=0.714) 95%CI(-4.075, -1.268), t=-3.743, p=<0.001	-2.19 (SE=0.4) 95%CI(-2.978, -1.403), t=-5.471, p=<0.001	-0.16 (SE=0.025) 95%CI(-0.21, -0.11), t=-6.338, p=<0.001	1.5 (SE=2.316) 95%CI(-3.107, 6.107), t=0.648, p=0.519	0.5 (SE=1.04) 95%CI(-1.569, 2.569), t=0.481, p=0.632	-1.7 (SE=1.032) 95%CI(-3.754, 0.354), t=-1.647, p=0.104	-6.4 (SE=0.725) 95%CI(-7.843, -4.957), t=-8.827, p=<0.001
T2-T4	0.722 (SE=0.1) 95%CI(0.525, 0.92), t=7.201, p=<0.001	-0.071 (SE=0.094) 95%CI(-0.256, 0.113), t=-0.761, p=0.447	-0.024 (SE=0.075) 95%CI(-0.171, 0.123), t=-0.319, p=0.75	0.024 (SE=0.294) 95%CI(-0.554, 0.602), t=0.081, p=0.936	-7.069 (SE=1.13) 95%CI(-9.292, -4.846), t=-6.253, p=<0.001	-3.274 (SE=0.641) 95%CI(-4.534, -2.014), t=-5.109, p=<0.001	-2.093 (SE=0.714) 95%CI(-3.496, -0.689), t=-2.932, p=0.004	-0.714 (SE=0.4) 95%CI(-1.502, 0.073), t=-1.784, p=0.075	-0.02 (SE=0.025) 95%CI(-0.07, 0.03), t=-0.792, p=0.431	-13.6 (SE=2.316) 95%CI(-18.207, -8.993), t=-5.873, p=<0.001	-1.9 (SE=1.04) 95%CI(-3.969, 0.169), t=-1.827, p=0.071	-2.4 (SE=1.032) 95%CI(-4.454, -0.346), t=-2.325, p=0.023	-1.3 (SE=0.725) 95%CI(-2.743, 0.143), t=-1.793, p=0.077
T2-T5	1.083 (SE=0.1) 95%CI(0.886, 1.281), t=10.802, p=<0.001	-0.024 (SE=0.094) 95%CI(-0.208, 0.161), t=-0.254, p=0.8	0.024 (SE=0.075) 95%CI(-0.123, 0.171), t=0.319, p=0.75	-0.005 (SE=0.294) 95%CI(-0.583, 0.573), t=-0.016, p=0.987	-0.379 (SE=1.13) 95%CI(-2.601, 1.844), t=-0.335, p=0.738	-1.155 (SE=0.641) 95%CI(-2.415, 0.105), t=-1.802, p=0.072	1.438 (SE=0.714) 95%CI(0.035, 2.842), t=2.015, p=0.045	-1.952 (SE=0.4) 95%CI(-2.74, -1.165), t=-4.876, p=<0.001	-0.02 (SE=0.025) 95%CI(-0.07, 0.03), t=-0.792, p=0.431	9.8 (SE=2.316) 95%CI(5.193, 14.407), t=4.232, p=<0.001	4.7 (SE=1.04) 95%CI(2.631, 6.769), t=4.519, p=<0.001	5.1 (SE=1.032) 95%CI(3.046, 7.154), t=4.94, p=<0.001	-6.2 (SE=0.725) 95%CI(-7.643, -4.757), t=-8.551, p=<0.001
T2-T6	1.556 (SE=0.1) 95%CI(1.358, 1.753), t=15.51, p=<0.001	-0.071 (SE=0.094) 95%CI(-0.256, 0.113), t=-0.761, p=0.447	-0.048 (SE=0.075) 95%CI(-0.194, 0.099), t=-0.638, p=0.524	-0.514 (SE=0.294) 95%CI(-1.092, 0.064), t=-1.749, p=0.081	-2.093 (SE=1.13) 95%CI(-4.316, 0.13), t=-1.851, p=0.065	-1.869 (SE=0.641) 95%CI(-3.129, -0.609), t=-2.917, p=0.004	-1.052 (SE=0.714) 95%CI(-2.456, 0.351), t=-1.474, p=0.141	-2.095 (SE=0.4) 95%CI(-2.883, -1.308), t=-5.233, p=<0.001	-0.16 (SE=0.025) 95%CI(-0.21, -0.11), t=-6.338, p=<0.001	6.2 (SE=2.316) 95%CI(1.593, 10.807), t=2.678, p=0.009	0.4 (SE=1.04) 95%CI(-1.669, 2.469), t=0.385, p=0.702	-3 (SE=1.032) 95%CI(-5.054, -0.946), t=-2.906, p=0.005	-7 (SE=0.725) 95%CI(-8.443, -5.557), t=-9.655, p=<0.001
T2-T7	1.889 (SE=0.1) 95%CI(1.692, 2.086), t=18.834, p=<0.001	-0.071 (SE=0.094) 95%CI(-0.256, 0.113), t=-0.761, p=0.447	-0.143 (SE=0.075) 95%CI(-0.29, 0.004), t=-1.913, p=0.057	-0.029 (SE=0.294) 95%CI(-0.607, 0.55), t=-0.097, p=0.923	-3.902 (SE=1.13) 95%CI(-6.125, -1.679), t=-3.452, p=0.001	-0.226 (SE=0.641) 95%CI(-1.486, 1.034), t=-0.353, p=0.724	0.669 (SE=0.714) 95%CI(-0.734, 2.073), t=0.937, p=0.349	-0.762 (SE=0.4) 95%CI(-1.549, 0.025), t=-1.903, p=0.058	-0.16 (SE=0.025) 95%CI(-0.21, -0.11), t=-6.338, p=<0.001	-5.2 (SE=2.316) 95%CI(-9.807, -0.593), t=-2.246, p=0.027	-0.4 (SE=1.04) 95%CI(-2.469, 1.669), t=-0.385, p=0.702	-2.1 (SE=1.032) 95%CI(-4.154, -0.046), t=-2.034, p=0.045	-1.4 (SE=0.725) 95%CI(-2.843, 0.043), t=-1.931, p=0.057
T2-T8	2.472 (SE=0.1) 95%CI(2.275, 2.67), t=24.65, p=<0.001	-0.19 (SE=0.094) 95%CI(-0.375, -0.006), t=-2.03, p=0.043	-0.19 (SE=0.075) 95%CI(-0.337, -0.044), t=-2.551, p=0.011	-0.005 (SE=0.294) 95%CI(-0.583, 0.573), t=-0.016, p=0.987	-4.093 (SE=1.13) 95%CI(-6.316, -1.87), t=-3.621, p=<0.001	-0.324 (SE=0.641) 95%CI(-1.584, 0.936), t=-0.505, p=0.614	-0.076 (SE=0.714) 95%CI(-1.48, 1.327), t=-0.107, p=0.915	-1.762 (SE=0.4) 95%CI(-2.549, -0.975), t=-4.4, p=<0.001	-0.13 (SE=0.025) 95%CI(-0.18, -0.08), t=-5.15, p=<0.001	-7.7 (SE=2.316) 95%CI(-12.307, -3.093), t=-3.325, p=0.001	1.7 (SE=1.04) 95%CI(-0.369, 3.769), t=1.635, p=0.106	-0.1 (SE=1.032) 95%CI(-2.154, 1.954), t=-0.097, p=0.923	-5.6 (SE=0.725) 95%CI(-7.043, -4.157), t=-7.724, p=<0.001
T2-T9	2.75 (SE=0.1) 95%CI(2.553, 2.947), t=27.42, p=<0.001	-0.19 (SE=0.094) 95%CI(-0.375, -0.006), t=-2.03, p=0.043	-0.262 (SE=0.075) 95%CI(-0.409, -0.115), t=-3.507, p=0.001	0.024 (SE=0.294) 95%CI(-0.554, 0.602), t=0.081, p=0.936	-4.64 (SE=1.13) 95%CI(-6.863, -2.418), t=-4.105, p=<0.001	-2.679 (SE=0.641) 95%CI(-3.939, -1.419), t=-4.18, p=<0.001	-1.514 (SE=0.714) 95%CI(-2.918, -0.111), t=-2.122, p=0.035	-1.929 (SE=0.4) 95%CI(-2.716, -1.141), t=-4.817, p=<0.001	-0.02 (SE=0.025) 95%CI(-0.07, 0.03), t=-0.792, p=0.431	-3.8 (SE=2.316) 95%CI(-8.407, 0.807), t=-1.641, p=0.105	-3.7 (SE=1.04) 95%CI(-5.769, -1.631), t=-3.558, p=0.001	-8 (SE=1.032) 95%CI(-10.054, -5.946), t=-7.748, p=<0.001	-6.3 (SE=0.725) 95%CI(-7.743, -4.857), t=-8.689, p=<0.001
T3-T10	2.611 (SE=0.1) 95%CI(2.414, 2.808), t=26.035, p=<0.001	0.071 (SE=0.094) 95%CI(-0.113, 0.256), t=0.761, p=0.447	-0.31 (SE=0.075) 95%CI(-0.456, -0.163), t=-	0.043 (SE=0.294) 95%CI(-0.535, 0.621), t=0.146, p=0.884	-2.738 (SE=1.13) 95%CI(-4.961, -0.515), t=-2.422, p=0.016	-0.214 (SE=0.641) 95%CI(-1.474, 1.046), t=-0.334, p=0.738	0.905 (SE=0.714) 95%CI(-0.499, 2.308), t=1.268, p=0.206	0.262 (SE=0.4) 95%CI(-0.525, 1.049), t=0.654, p=0.513	0.14 (SE=0.025) 95%CI(0.09, 0.19), t=5.546, p=<0.001	-7.5 (SE=2.316) 95%CI(-12.107, -2.893), t=-3.239, p=0.002	-4.9 (SE=1.04) 95%CI(-6.969, -2.831), t=-	-6 (SE=1.032) 95%CI(-8.054, -3.946), t=-5.811, p=<0.001	0.1 (SE=0.725) 95%CI(-1.343, 1.543), t=0.138, p=0.891

			4.145, p=<0.001								4.712, p=<0.001		
T3-T4	0.5 (SE=0.1) 95%CI(0.303, 0.697), t=4.985, p=<0.001	0.119 (SE=0.094) 95%CI(-0.065, 0.304), t=1.269, p=0.205	-0.048 (SE=0.075) 95%CI(-0.194, 0.099), t=-0.638, p=0.524	0.038 (SE=0.294) 95%CI(-0.54, 0.616), t=0.13, p=0.897	-5.119 (SE=1.13) 95%CI(-7.342, -2.896), t=-4.528, p=<0.001	-1.476 (SE=0.641) 95%CI(-2.736, -0.216), t=-2.304, p=0.022	0.579 (SE=0.714) 95%CI(-0.825, 1.982), t=0.811, p=0.418	1.476 (SE=0.4) 95%CI(0.689, 2.264), t=3.687, p=<0.001	0.14 (SE=0.025) 95%CI(0.09, 0.19), t=5.546, p=<0.001	-15.1 (SE=2.316) 95%CI(-19.707, -10.493), t=-6.521, p=<0.001	-2.4 (SE=1.04) 95%CI(-4.469, -0.331), t=-2.308, p=0.024	-0.7 (SE=1.032) 95%CI(-2.754, 1.354), t=-0.678, p=0.5	5.1 (SE=0.725) 95%CI(3.657, 6.543), t=7.034, p=<0.001
T3-T5	0.861 (SE=0.1) 95%CI(0.664, 1.058), t=8.586, p=<0.001	0.167 (SE=0.094) 95%CI(-0.018, 0.351), t=1.777, p=0.076	0 (SE=0.075) 95%CI(-0.147, 0.147), t=0, p=>0.999	0.01 (SE=0.294) 95%CI(-0.569, 0.588), t=0.032, p=0.974	1.571 (SE=1.13) 95%CI(-0.651, 3.794), t=1.39, p=0.165	0.643 (SE=0.641) 95%CI(-0.617, 1.903), t=1.003, p=0.316	4.11 (SE=0.714) 95%CI(2.706, 5.513), t=5.758, p=<0.001	0.238 (SE=0.4) 95%CI(-0.549, 1.025), t=0.595, p=0.552	0.14 (SE=0.025) 95%CI(0.09, 0.19), t=5.546, p=<0.001	8.3 (SE=2.316) 95%CI(3.693, 12.907), t=3.585, p=0.001	4.2 (SE=1.04) 95%CI(2.131, 6.269), t=4.039, p=<0.001	6.8 (SE=1.032) 95%CI(4.746, 8.854), t=6.586, p=<0.001	0.2 (SE=0.725) 95%CI(-1.243, 1.643), t=0.276, p=0.783
T3-T6	1.333 (SE=0.1) 95%CI(1.136, 1.531), t=13.294, p=<0.001	0.119 (SE=0.094) 95%CI(-0.065, 0.304), t=1.269, p=0.205	-0.071 (SE=0.075) 95%CI(-0.218, 0.075), t=-0.957, p=0.339	-0.5 (SE=0.294) 95%CI(-1.078, 0.078), t=-1.701, p=0.09	-0.143 (SE=1.13) 95%CI(-2.366, 2.08), t=-0.126, p=0.9	-0.071 (SE=0.641) 95%CI(-1.331, 1.189), t=-0.111, p=0.911	1.619 (SE=0.714) 95%CI(0.216, 3.023), t=2.268, p=0.024	0.095 (SE=0.4) 95%CI(-0.692, 0.883), t=0.238, p=0.812	0 (SE=0.025) 95%CI(-0.05, 0.05), t=0, p=>0.999	4.7 (SE=2.316) 95%CI(0.093, 9.307), t=2.03, p=0.046	-0.1 (SE=1.04) 95%CI(-2.169, 1.969), t=-0.096, p=0.924	-1.3 (SE=1.032) 95%CI(-3.354, 0.754), t=-1.259, p=0.212	-0.6 (SE=0.725) 95%CI(-2.043, 0.843), t=-0.828, p=0.41
T3-T7	1.667 (SE=0.1) 95%CI(1.469, 1.864), t=16.618, p=<0.001	0.119 (SE=0.094) 95%CI(-0.065, 0.304), t=1.269, p=0.205	-0.167 (SE=0.075) 95%CI(-0.314, -0.02), t=-2.232, p=0.026	-0.014 (SE=0.294) 95%CI(-0.592, 0.564), t=-0.049, p=0.961	-1.952 (SE=1.13) 95%CI(-4.175, 0.271), t=-1.727, p=0.085	1.571 (SE=0.641) 95%CI(0.311, 2.831), t=2.452, p=0.015	3.34 (SE=0.714) 95%CI(1.937, 4.744), t=4.68, p=<0.001	1.429 (SE=0.4) 95%CI(0.641, 2.216), t=3.568, p=<0.001	0 (SE=0.025) 95%CI(-0.05, 0.05), t=0, p=>0.999	-6.7 (SE=2.316) 95%CI(-11.307, -2.093), t=-2.894, p=0.005	-0.9 (SE=1.04) 95%CI(-2.969, 1.169), t=-0.865, p=0.389	-0.4 (SE=1.032) 95%CI(-2.454, 1.654), t=-0.387, p=0.699	5 (SE=0.725) 95%CI(3.557, 6.443), t=6.896, p=<0.001
T3-T8	2.25 (SE=0.1) 95%CI(2.053, 2.447), t=22.434, p=<0.001	0 (SE=0.094) 95%CI(-0.184, 0.184), t=0, p=>0.999	-0.214 (SE=0.075) 95%CI(-0.361, -0.067), t=-2.87, p=0.004	0.01 (SE=0.294) 95%CI(-0.569, 0.588), t=0.032, p=0.974	-2.143 (SE=1.13) 95%CI(-4.366, 0.08), t=-1.896, p=0.059	1.474 (SE=0.641) 95%CI(0.214, 2.734), t=2.3, p=0.022	2.595 (SE=0.714) 95%CI(1.192, 3.999), t=3.636, p=<0.001	0.429 (SE=0.4) 95%CI(-0.359, 1.216), t=1.07, p=0.285	0.03 (SE=0.025) 95%CI(-0.02, 0.08), t=1.188, p=0.238	-9.2 (SE=2.316) 95%CI(-13.807, -4.593), t=-3.973, p=<0.001	1.2 (SE=1.04) 95%CI(-0.869, 3.269), t=1.154, p=0.252	1.6 (SE=1.032) 95%CI(-0.454, 3.654), t=1.55, p=0.125	0.8 (SE=0.725) 95%CI(-0.643, 2.243), t=1.103, p=0.273
T3-T9	2.528 (SE=0.1) 95%CI(2.33, 2.725), t=25.204, p=<0.001	0 (SE=0.094) 95%CI(-0.184, 0.184), t=0, p=>0.999	-0.286 (SE=0.075) 95%CI(-0.433, -0.139), t=-3.826, p=<0.001	0.038 (SE=0.294) 95%CI(-0.54, 0.616), t=0.13, p=0.897	-2.69 (SE=1.13) 95%CI(-4.913, -0.468), t=-2.38, p=0.018	-0.881 (SE=0.641) 95%CI(-2.141, 0.379), t=-1.375, p=0.17	1.157 (SE=0.714) 95%CI(-0.246, 2.561), t=1.621, p=0.106	0.262 (SE=0.4) 95%CI(-0.525, 1.049), t=0.654, p=0.513	0.14 (SE=0.025) 95%CI(0.09, 0.19), t=5.546, p=<0.001	-5.3 (SE=2.316) 95%CI(-9.907, -0.693), t=-2.289, p=0.025	-4.2 (SE=1.04) 95%CI(-6.269, -2.131), t=-4.039, p=<0.001	-6.3 (SE=1.032) 95%CI(-8.354, -4.246), t=-6.102, p=<0.001	0.1 (SE=0.725) 95%CI(-1.343, 1.543), t=0.138, p=0.891
T4-T10	2.111 (SE=0.1) 95%CI(1.914, 2.308), t=21.049, p=<0.001	-0.048 (SE=0.094) 95%CI(-0.232, 0.137), t=-0.508, p=0.612	-0.262 (SE=0.075) 95%CI(-0.409, -0.115), t=-3.507, p=0.001	0.005 (SE=0.294) 95%CI(-0.573, 0.583), t=0.016, p=0.987	2.381 (SE=1.13) 95%CI(0.158, 4.604), t=2.106, p=0.036	1.262 (SE=0.641) 95%CI(0.002, 2.522), t=1.969, p=0.05	0.326 (SE=0.714) 95%CI(-1.077, 1.73), t=0.457, p=0.648	-1.214 (SE=0.4) 95%CI(-2.002, -0.427), t=-3.033, p=0.003	0 (SE=0.025) 95%CI(-0.05, 0.05), t=0, p=>0.999	7.6 (SE=2.316) 95%CI(2.993, 12.207), t=3.282, p=0.002	-2.5 (SE=1.04) 95%CI(-4.569, -0.431), t=-2.404, p=0.019	-5.3 (SE=1.032) 95%CI(-7.354, -3.246), t=-5.133, p=<0.001	-5 (SE=0.725) 95%CI(-6.443, -3.557), t=-6.896, p=<0.001
T4-T5	0.361 (SE=0.1) 95%CI(0.164, 0.558), t=3.601, p=<0.001	0.048 (SE=0.094) 95%CI(-0.137, 0.232), t=0.508, p=0.612	0.048 (SE=0.075) 95%CI(-0.099, 0.194), t=0.638, p=0.524	-0.029 (SE=0.294) 95%CI(-0.607, 0.55), t=-0.097, p=0.923	6.69 (SE=1.13) 95%CI(4.468, 8.913), t=5.919, p=<0.001	2.119 (SE=0.641) 95%CI(0.859, 3.379), t=3.307, p=0.001	3.531 (SE=0.714) 95%CI(2.127, 4.934), t=4.947, p=<0.001	-1.238 (SE=0.4) 95%CI(-2.025, -0.451), t=-3.092, p=0.002	0 (SE=0.025) 95%CI(-0.05, 0.05), t=0, p=>0.999	23.4 (SE=2.316) 95%CI(18.793, 28.007), t=10.106, p=<0.001	6.6 (SE=1.04) 95%CI(4.531, 8.669), t=6.346, p=<0.001	7.5 (SE=1.032) 95%CI(5.446, 9.554), t=7.264, p=<0.001	-4.9 (SE=0.725) 95%CI(-6.343, -3.457), t=-6.758, p=<0.001
T4-T6	0.833 (SE=0.1) 95%CI(0.636, 1.031), t=8.309, p=<0.001	0 (SE=0.094) 95%CI(-0.184, 0.184), t=0, p=>0.999	-0.024 (SE=0.075) 95%CI(-0.171, 0.123), t=-0.319, p=0.75	-0.538 (SE=0.294) 95%CI(-1.116, 0.04), t=-1.83, p=0.068	4.976 (SE=1.13) 95%CI(2.753, 7.199), t=4.402, p=<0.001	1.405 (SE=0.641) 95%CI(0.145, 2.665), t=2.192, p=0.029	1.04 (SE=0.714) 95%CI(-0.363, 2.444), t=1.458, p=0.146	-1.381 (SE=0.4) 95%CI(-2.168, -0.594), t=-3.449, p=0.001	-0.14 (SE=0.025) 95%CI(-0.19, -0.09), t=-5.546, p=<0.001	19.8 (SE=2.316) 95%CI(15.193, 24.407), t=8.551, p=<0.001	2.3 (SE=1.04) 95%CI(0.231, 4.369), t=2.212, p=0.03	-0.6 (SE=1.032) 95%CI(-2.654, 1.454), t=-0.581, p=0.563	-5.7 (SE=0.725) 95%CI(-7.143, -4.257), t=-7.862, p=<0.001
T4-T7	1.167 (SE=0.1) 95%CI(0.969, 1.364),	0 (SE=0.094) 95%CI(-0.184, 0.184), t=0, p=>0.999	-0.119 (SE=0.075) 95%CI(-0.266, 0.028),	-0.052 (SE=0.294) 95%CI(-0.631, 0.526),	3.167 (SE=1.13) 95%CI(0.944,	3.048 (SE=0.641) 95%CI(1.788, 4.308),	2.762 (SE=0.714) 95%CI(1.358, 4.165),	-0.048 (SE=0.4) 95%CI(-0.835, 0.74),	-0.14 (SE=0.025) 95%CI(-0.19, -0.09), t=-	8.4 (SE=2.316) 95%CI(3.793, 13.007),	1.5 (SE=1.04) 95%CI(-0.569, 3.569),	0.3 (SE=1.032) 95%CI(-1.754, 2.354),	-0.1 (SE=0.725) 95%CI(-1.543, 1.343), t=-0.138, p=0.891

	t=11.633, p=<0.001		t=-1.594, p=0.112	t=-0.178, p=0.859	5.39), t=2.801, p=0.005	t=4.756, p=<0.001	t=3.87, p=<0.001	t=-0.119, p=0.905	5.546, p=<0.001	t=3.628, p=<0.001	t=1.442, p=0.153	t=0.291, p=0.772	
T4-T8	1.75 (SE=0.1) 95%CI(1.553, 1.947), t=17.449, p=<0.001	-0.119 (SE=0.094) 95%CI(-0.304, 0.065), t=-1.269, p=0.205	-0.167 (SE=0.075) 95%CI(-0.314, -0.02), t=-2.232, p=0.026	-0.029 (SE=0.294) 95%CI(-0.607, 0.55), t=-0.097, p=0.923	2.976 (SE=1.13) 95%CI(0.753, 5.199), t=2.633, p=0.009	2.95 (SE=0.641) 95%CI(1.69, 4.21), t=4.604, p=<0.001	2.017 (SE=0.714) 95%CI(0.613, 3.42), t=2.826, p=0.005	-1.048 (SE=0.4) 95%CI(-1.835, -0.26), t=-2.616, p=0.009	-0.11 (SE=0.025) 95%CI(-0.16, -0.06), t=-4.357, p=<0.001	5.9 (SE=2.316) 95%CI(1.293, 10.507), t=2.548, p=0.013	3.6 (SE=1.04) 95%CI(1.531, 5.669), t=3.462, p=0.001	2.3 (SE=1.032) 95%CI(0.246, 4.354), t=2.228, p=0.029	-4.3 (SE=0.725) 95%CI(-5.743, -2.857), t=-5.931, p=<0.001
T4-T9	2.028 (SE=0.1) 95%CI(1.83, 2.225), t=20.218, p=<0.001	-0.119 (SE=0.094) 95%CI(-0.304, 0.065), t=-1.269, p=0.205	-0.238 (SE=0.075) 95%CI(-0.385, -0.091), t=-3.188, p=0.002	0 (SE=0.294) 95%CI(-0.578, 0.578), t=0, p=>0.999	2.429 (SE=1.13) 95%CI(0.206, 4.651), t=2.148, p=0.032	0.595 (SE=0.641) 95%CI(-0.665, 1.855), t=0.929, p=0.354	0.579 (SE=0.714) 95%CI(-0.825, 1.982), t=0.811, p=0.418	-1.214 (SE=0.4) 95%CI(-2.002, -0.427), t=-3.033, p=0.003	0 (SE=0.025) 95%CI(-0.05, 0.05), t=0, p=>0.999	9.8 (SE=2.316) 95%CI(5.193, 14.407), t=4.232, p=<0.001	-1.8 (SE=1.04) 95%CI(-3.869, 0.269), t=-1.731, p=0.087	-5.6 (SE=1.032) 95%CI(-7.654, -3.546), t=-5.424, p=<0.001	-5 (SE=0.725) 95%CI(-6.443, -3.557), t=-6.896, p=<0.001
T5-T10	1.75 (SE=0.1) 95%CI(1.553, 1.947), t=17.449, p=<0.001	-0.095 (SE=0.094) 95%CI(-0.28, 0.089), t=-1.015, p=0.311	-0.31 (SE=0.075) 95%CI(-0.456, -0.163), t=-4.145, p=<0.001	0.033 (SE=0.294) 95%CI(-0.545, 0.611), t=0.113, p=0.91	-4.31 (SE=1.13) 95%CI(-6.532, -2.087), t=-3.812, p=<0.001	-0.857 (SE=0.641) 95%CI(-2.117, 0.403), t=-1.338, p=0.182	-3.205 (SE=0.714) 95%CI(-4.608, -1.801), t=-4.49, p=<0.001	0.024 (SE=0.4) 95%CI(-0.764, 0.811), t=0.059, p=0.953	0 (SE=0.025) 95%CI(-0.05, 0.05), t=0, p=>0.999	-15.8 (SE=2.316) 95%CI(-20.407, -11.193), t=-6.824, p=<0.001	-9.1 (SE=1.04) 95%CI(-11.169, -7.031), t=-8.75, p=<0.001	-12.8 (SE=1.032) 95%CI(-14.854, -10.746), t=-12.397, p=<0.001	-0.1 (SE=0.725) 95%CI(-1.543, 1.343), t=-0.138, p=0.891
T5-T6	0.472 (SE=0.1) 95%CI(0.275, 0.67), t=4.708, p=<0.001	-0.048 (SE=0.094) 95%CI(-0.232, 0.137), t=-0.508, p=0.612	-0.071 (SE=0.075) 95%CI(-0.218, 0.075), t=-0.957, p=0.339	-0.51 (SE=0.294) 95%CI(-1.088, 0.069), t=-1.733, p=0.084	-1.714 (SE=1.13) 95%CI(-3.937, 0.509), t=-1.516, p=0.13	-0.714 (SE=0.641) 95%CI(-1.974, 0.546), t=-1.115, p=0.266	-2.49 (SE=0.714) 95%CI(-3.894, -1.087), t=-3.489, p=0.001	-0.143 (SE=0.4) 95%CI(-0.93, 0.644), t=-0.357, p=0.721	-0.14 (SE=0.025) 95%CI(-0.19, -0.09), t=-5.546, p=<0.001	-3.6 (SE=2.316) 95%CI(-8.207, 1.007), t=-1.555, p=0.124	-4.3 (SE=1.04) 95%CI(-6.369, -2.231), t=-4.135, p=<0.001	-8.1 (SE=1.032) 95%CI(-10.154, -6.046), t=-7.845, p=<0.001	-0.8 (SE=0.725) 95%CI(-2.243, 0.643), t=-1.103, p=0.273
T5-T7	0.806 (SE=0.1) 95%CI(0.608, 1.003), t=8.032, p=<0.001	-0.048 (SE=0.094) 95%CI(-0.232, 0.137), t=-0.508, p=0.612	-0.167 (SE=0.075) 95%CI(-0.314, -0.02), t=-2.232, p=0.026	-0.024 (SE=0.294) 95%CI(-0.602, 0.554), t=-0.081, p=0.936	-3.524 (SE=1.13) 95%CI(-5.747, -1.301), t=-3.117, p=0.002	0.929 (SE=0.641) 95%CI(-0.331, 2.189), t=1.449, p=0.148	-0.769 (SE=0.714) 95%CI(-2.173, 0.634), t=-1.078, p=0.282	1.19 (SE=0.4) 95%CI(0.403, 1.978), t=2.973, p=0.003	-0.14 (SE=0.025) 95%CI(-0.19, -0.09), t=-5.546, p=<0.001	-15 (SE=2.316) 95%CI(-19.607, -10.393), t=-6.478, p=<0.001	-5.1 (SE=1.04) 95%CI(-7.169, -3.031), t=-4.904, p=<0.001	-7.2 (SE=1.032) 95%CI(-9.254, -5.146), t=-6.974, p=<0.001	4.8 (SE=0.725) 95%CI(3.357, 6.243), t=6.621, p=<0.001
T5-T8	1.389 (SE=0.1) 95%CI(1.192, 1.586), t=13.848, p=<0.001	-0.167 (SE=0.094) 95%CI(-0.351, 0.018), t=-1.777, p=0.076	-0.214 (SE=0.075) 95%CI(-0.361, -0.067), t=-2.87, p=0.004	0 (SE=0.294) 95%CI(-0.578, 0.578), t=0, p=>0.999	-3.714 (SE=1.13) 95%CI(-5.937, -1.491), t=-3.286, p=0.001	0.831 (SE=0.641) 95%CI(-0.429, 2.091), t=1.297, p=0.196	-1.514 (SE=0.714) 95%CI(-2.918, -0.111), t=-2.122, p=0.035	0.19 (SE=0.4) 95%CI(-0.597, 0.978), t=0.476, p=0.635	-0.11 (SE=0.025) 95%CI(-0.16, -0.06), t=-4.357, p=<0.001	-17.5 (SE=2.316) 95%CI(-22.107, -12.893), t=-7.558, p=<0.001	-3 (SE=1.04) 95%CI(-5.069, -0.931), t=-2.885, p=0.005	-5.2 (SE=1.032) 95%CI(-7.254, -3.146), t=-5.036, p=<0.001	0.6 (SE=0.725) 95%CI(-0.843, 2.043), t=0.828, p=0.41
T5-T9	1.667 (SE=0.1) 95%CI(1.469, 1.864), t=16.618, p=<0.001	-0.167 (SE=0.094) 95%CI(-0.351, 0.018), t=-1.777, p=0.076	-0.286 (SE=0.075) 95%CI(-0.433, -0.139), t=-3.826, p=<0.001	0.029 (SE=0.294) 95%CI(-0.55, 0.607), t=0.097, p=0.923	-4.262 (SE=1.13) 95%CI(-6.485, -2.039), t=-3.77, p=<0.001	-1.524 (SE=0.641) 95%CI(-2.784, -0.264), t=-2.378, p=0.018	-2.952 (SE=0.714) 95%CI(-4.356, -1.549), t=-4.137, p=<0.001	0.024 (SE=0.4) 95%CI(-0.764, 0.811), t=0.059, p=0.953	0 (SE=0.025) 95%CI(-0.05, 0.05), t=0, p=>0.999	-13.6 (SE=2.316) 95%CI(-18.207, -8.993), t=-5.873, p=<0.001	-8.4 (SE=1.04) 95%CI(-10.469, -6.331), t=-8.077, p=<0.001	-13.1 (SE=1.032) 95%CI(-15.154, -11.046), t=-12.688, p=<0.001	-0.1 (SE=0.725) 95%CI(-1.543, 1.343), t=-0.138, p=0.891
T6-T10	1.278 (SE=0.1) 95%CI(1.08, 1.475), t=12.74, p=<0.001	-0.048 (SE=0.094) 95%CI(-0.232, 0.137), t=-0.508, p=0.612	-0.238 (SE=0.075) 95%CI(-0.385, -0.091), t=-3.188, p=0.002	0.543 (SE=0.294) 95%CI(-0.035, 1.121), t=1.846, p=0.066	-2.595 (SE=1.13) 95%CI(-4.818, -0.372), t=-2.296, p=0.022	-0.143 (SE=0.641) 95%CI(-1.403, 1.117), t=-0.223, p=0.824	-0.714 (SE=0.714) 95%CI(-2.118, 0.689), t=-1.001, p=0.318	0.167 (SE=0.4) 95%CI(-0.621, 0.954), t=0.416, p=0.677	0.14 (SE=0.025) 95%CI(0.09, 0.19), t=5.546, p=<0.001	-12.2 (SE=2.316) 95%CI(-16.807, -7.593), t=-5.269, p=<0.001	-4.8 (SE=1.04) 95%CI(-6.869, -2.731), t=-4.616, p=<0.001	-4.7 (SE=1.032) 95%CI(-6.754, -2.646), t=-4.552, p=<0.001	0.7 (SE=0.725) 95%CI(-0.743, 2.143), t=0.965, p=0.337
T6-T7	0.333 (SE=0.1) 95%CI(0.136, 0.531), t=3.324, p=0.001	0 (SE=0.094) 95%CI(-0.184, 0.184), t=0, p=>0.999	-0.095 (SE=0.075) 95%CI(-0.242, 0.052), t=-1.275, p=0.203	0.486 (SE=0.294) 95%CI(-0.092, 1.064), t=1.652, p=0.099	-1.81 (SE=1.13) 95%CI(-4.032, 0.413), t=-1.601, p=0.11	1.643 (SE=0.641) 95%CI(0.383, 2.903), t=2.564, p=0.011	1.721 (SE=0.714) 95%CI(0.318, 3.125), t=2.412, p=0.016	1.333 (SE=0.4) 95%CI(0.546, 2.121), t=3.33, p=0.001	0 (SE=0.025) 95%CI(-0.05, 0.05), t=0, p=>0.999	-11.4 (SE=2.316) 95%CI(-16.007, -6.793), t=-4.923, p=<0.001	-0.8 (SE=1.04) 95%CI(-2.869, 1.269), t=-0.769, p=0.444	0.9 (SE=1.032) 95%CI(-1.154, 2.954), t=0.872, p=0.386	5.6 (SE=0.725) 95%CI(4.157, 7.043), t=7.724, p=<0.001

T6-T8	0.917 (SE=0.1) 95%CI(0.719, 1.114), t=9.14, p=<0.001	-0.119 (SE=0.094) 95%CI(-0.304, 0.065), t=-1.269, p=0.205	-0.143 (SE=0.075) 95%CI(-0.29, 0.004), t=-1.913, p=0.057	0.51 (SE=0.294) 95%CI(-0.069, 1.088), t=1.733, p=0.084	-2 (SE=1.13) 95%CI(-4.223, 0.223), t=-1.769, p=0.078	1.545 (SE=0.641) 95%CI(0.285, 2.805), t=2.412, p=0.016	0.976 (SE=0.714) 95%CI(-0.427, 2.38), t=1.368, p=0.172	0.333 (SE=0.4) 95%CI(-0.454, 1.121), t=0.833, p=0.406	0.03 (SE=0.025) 95%CI(-0.02, 0.08), t=1.188, p=0.238	-13.9 (SE=2.316) 95%CI(-18.507, -9.293), t=-6.003, p=<0.001	1.3 (SE=1.04) 95%CI(-0.769, 3.369), t=1.25, p=0.215	2.9 (SE=1.032) 95%CI(0.846, 4.954), t=2.809, p=0.006	1.4 (SE=0.725) 95%CI(-0.043, 2.843), t=1.931, p=0.057
T6-T9	1.194 (SE=0.1) 95%CI(0.997, 1.392), t=11.909, p=<0.001	-0.119 (SE=0.094) 95%CI(-0.304, 0.065), t=-1.269, p=0.205	-0.214 (SE=0.075) 95%CI(-0.361, -0.067), t=-2.87, p=0.004	0.538 (SE=0.294) 95%CI(-0.04, 1.116), t=1.83, p=0.068	-2.548 (SE=1.13) 95%CI(-4.771, -0.325), t=-2.254, p=0.025	-0.81 (SE=0.641) 95%CI(-2.07, 0.45), t=-1.263, p=0.207	-0.462 (SE=0.714) 95%CI(-1.865, 0.942), t=-0.647, p=0.518	0.167 (SE=0.4) 95%CI(-0.621, 0.954), t=0.416, p=0.677	0.14 (SE=0.025) 95%CI(0.09, 0.19), t=5.546, p=<0.001	-10 (SE=2.316) 95%CI(-14.607, -5.393), t=-4.319, p=<0.001	-4.1 (SE=1.04) 95%CI(-6.169, -2.031), t=-3.943, p=<0.001	-5 (SE=1.032) 95%CI(-7.054, -2.946), t=-4.843, p=<0.001	0.7 (SE=0.725) 95%CI(-0.743, 2.143), t=0.965, p=0.337
T7-T10	0.944 (SE=0.1) 95%CI(0.747, 1.142), t=9.417, p=<0.001	-0.048 (SE=0.094) 95%CI(-0.232, 0.137), t=-0.508, p=0.612	-0.143 (SE=0.075) 95%CI(-0.29, 0.004), t=-1.913, p=0.057	0.057 (SE=0.294) 95%CI(-0.521, 0.635), t=0.194, p=0.846	-0.786 (SE=1.13) 95%CI(-3.009, 1.437), t=-0.695, p=0.487	-1.786 (SE=0.641) 95%CI(-3.046, -0.526), t=-2.787, p=0.006	-2.436 (SE=0.714) 95%CI(-3.839, -1.032), t=-3.413, p=0.001	-1.167 (SE=0.4) 95%CI(-1.954, -0.379), t=-2.914, p=0.004	0.14 (SE=0.025) 95%CI(0.09, 0.19), t=5.546, p=<0.001	-0.8 (SE=2.316) 95%CI(-5.407, 3.807), t=-0.345, p=0.731	-4 (SE=1.04) 95%CI(-6.069, -1.931), t=-3.846, p=<0.001	-5.6 (SE=1.032) 95%CI(-7.654, -3.546), t=-5.424, p=<0.001	-4.9 (SE=0.725) 95%CI(-6.343, -3.457), t=-6.758, p=<0.001
T7-T8	0.583 (SE=0.1) 95%CI(0.386, 0.781), t=5.816, p=<0.001	-0.119 (SE=0.094) 95%CI(-0.304, 0.065), t=-1.269, p=0.205	-0.048 (SE=0.075) 95%CI(-0.194, 0.099), t=-0.638, p=0.524	0.024 (SE=0.294) 95%CI(-0.554, 0.602), t=0.081, p=0.936	-0.19 (SE=1.13) 95%CI(-2.413, 2.032), t=-0.168, p=0.866	-0.098 (SE=0.641) 95%CI(-1.358, 1.162), t=-0.152, p=0.879	-0.745 (SE=0.714) 95%CI(-2.149, 0.658), t=-1.044, p=0.297	-1 (SE=0.4) 95%CI(-1.787, -0.213), t=-2.498, p=0.013	0.03 (SE=0.025) 95%CI(-0.02, 0.08), t=1.188, p=0.238	-2.5 (SE=2.316) 95%CI(-7.107, 2.107), t=-1.08, p=0.283	2.1 (SE=1.04) 95%CI(0.031, 4.169), t=2.019, p=0.047	2 (SE=1.032) 95%CI(-0.054, 4.054), t=1.937, p=0.056	-4.2 (SE=0.725) 95%CI(-5.643, -2.757), t=-5.793, p=<0.001
T7-T9	0.861 (SE=0.1) 95%CI(0.664, 1.058), t=8.586, p=<0.001	-0.119 (SE=0.094) 95%CI(-0.304, 0.065), t=-1.269, p=0.205	-0.119 (SE=0.075) 95%CI(-0.266, 0.028), t=-1.594, p=0.112	0.052 (SE=0.294) 95%CI(-0.526, 0.631), t=0.178, p=0.859	-0.738 (SE=1.13) 95%CI(-2.961, 1.485), t=-0.653, p=0.514	-2.452 (SE=0.641) 95%CI(-3.712, -1.192), t=-3.827, p=<0.001	-2.183 (SE=0.714) 95%CI(-3.587, -0.78), t=-3.059, p=0.002	-1.167 (SE=0.4) 95%CI(-1.954, -0.379), t=-2.914, p=0.004	0.14 (SE=0.025) 95%CI(0.09, 0.19), t=5.546, p=<0.001	1.4 (SE=2.316) 95%CI(-3.207, 6.007), t=0.605, p=0.547	-3.3 (SE=1.04) 95%CI(-5.369, -1.231), t=-3.173, p=0.002	-5.9 (SE=1.032) 95%CI(-7.954, -3.846), t=-5.714, p=<0.001	-4.9 (SE=0.725) 95%CI(-6.343, -3.457), t=-6.758, p=<0.001
T8-T10	0.361 (SE=0.1) 95%CI(0.164, 0.558), t=3.601, p=<0.001	0.071 (SE=0.094) 95%CI(-0.113, 0.256), t=0.761, p=0.447	-0.095 (SE=0.075) 95%CI(-0.242, 0.052), t=-1.275, p=0.203	0.033 (SE=0.294) 95%CI(-0.545, 0.611), t=0.113, p=0.91	-0.595 (SE=1.13) 95%CI(-2.818, 1.628), t=-0.527, p=0.599	-1.688 (SE=0.641) 95%CI(-2.948, -0.428), t=-2.635, p=0.009	-1.69 (SE=0.714) 95%CI(-3.094, -0.287), t=-2.369, p=0.018	-0.167 (SE=0.4) 95%CI(-0.954, 0.621), t=-0.416, p=0.677	0.11 (SE=0.025) 95%CI(0.06, 0.16), t=4.357, p=<0.001	1.7 (SE=2.316) 95%CI(-2.907, 6.307), t=0.734, p=0.465	-6.1 (SE=1.04) 95%CI(-8.169, -4.031), t=-5.866, p=<0.001	-7.6 (SE=1.032) 95%CI(-9.654, -5.546), t=-7.361, p=<0.001	-0.7 (SE=0.725) 95%CI(-2.143, 0.743), t=-0.965, p=0.337
T8-T9	0.278 (SE=0.1) 95%CI(0.08, 0.475), t=2.77, p=0.006	0 (SE=0.094) 95%CI(-0.184, 0.184), t=0, p=>0.999	-0.071 (SE=0.075) 95%CI(-0.218, 0.075), t=-0.957, p=0.339	0.029 (SE=0.294) 95%CI(-0.55, 0.607), t=0.097, p=0.923	-0.548 (SE=1.13) 95%CI(-2.771, 1.675), t=-0.484, p=0.628	-2.355 (SE=0.641) 95%CI(-3.615, -1.095), t=-3.675, p=<0.001	-1.438 (SE=0.714) 95%CI(-2.842, -0.035), t=-2.015, p=0.045	-0.167 (SE=0.4) 95%CI(-0.954, 0.621), t=-0.416, p=0.677	0.11 (SE=0.025) 95%CI(0.06, 0.16), t=4.357, p=<0.001	3.9 (SE=2.316) 95%CI(-0.707, 8.507), t=1.684, p=0.096	-5.4 (SE=1.04) 95%CI(-7.469, -3.331), t=-5.193, p=<0.001	-7.9 (SE=1.032) 95%CI(-9.954, -5.846), t=-7.652, p=<0.001	-0.7 (SE=0.725) 95%CI(-2.143, 0.743), t=-0.965, p=0.337
T9-T10	0.083 (SE=0.1) 95%CI(-0.114, 0.281), t=0.831, p=0.407	0.071 (SE=0.094) 95%CI(-0.113, 0.256), t=0.761, p=0.447	-0.024 (SE=0.075) 95%CI(-0.171, 0.123), t=-0.319, p=0.75	0.005 (SE=0.294) 95%CI(-0.573, 0.583), t=0.016, p=0.987	-0.048 (SE=1.13) 95%CI(-2.271, 2.175), t=-0.042, p=0.966	0.667 (SE=0.641) 95%CI(-0.593, 1.927), t=1.04, p=0.299	-0.252 (SE=0.714) 95%CI(-1.656, 1.151), t=-0.354, p=0.724	0 (SE=0.4) 95%CI(-0.787, 0.787), t=0, p=>0.999	0 (SE=0.025) 95%CI(-0.05, 0.05), t=0, p=>0.999	-2.2 (SE=2.316) 95%CI(-6.807, 2.407), t=-0.95, p=0.345	-0.7 (SE=1.04) 95%CI(-2.769, 1.369), t=-0.673, p=0.503	0.3 (SE=1.032) 95%CI(-1.754, 2.354), t=0.291, p=0.772	0 (SE=0.725) 95%CI(-1.443, 1.443), t=0, p=>0.999

(SE): model coefficient (standard error); 95%CI: 95% confidence interval; t: Significant if $p<0.05$ (shown in red).

Supplementary material. Table 4: Radiofrequency parameters between both legs model coefficient terms results.

	Frequency (Hz)	Maximum power (Watts)	Average power (Watts)	Absorved energy (Volt-ampere)	Temperature (Celsius)
(Intercept)	1.17 (SE=0.113) 95%CI(0.938, 1.405), t=10.373, p=<0.001	11.224 (SE=1.877) 95%CI(7.745, 14.74), t=5.98, p=<0.001	6.137 (SE=0.539) 95%CI(5.109, 7.302), t=11.384, p=<0.001	4.956 (SE=0.857) 95%CI(3.598, 6.409), t=5.781, p=<0.001	35.203 (SE=1.632) 95%CI(31.342, 39.169), t=21.566, p=<0.001
Group (left)	-0.14 (SE=0.25) 95%CI(-0.72, 0.542), t=-0.558, p=0.577	0.756 (SE=1.863) 95%CI(-3.151, 4.054), t=0.406, p=0.685	1.654 (SE=1.011) 95%CI(-0.337, 3.719), t=1.635, p=0.103	-1.766 (SE=1.149) 95%CI(-3.971, 0.343), t=-1.536, p=0.125	-3.354 (SE=0.575) 95%CI(-4.378, -2.022), t=-5.834, p=<0.001
Group (left):T3	0.146 (SE=0.351) 95%CI(-0.595, 1.035), t=0.415, p=0.679	-3.45 (SE=2.56) 95%CI(-8.937, 1.134), t=-1.347, p=0.179	-2.298 (SE=1.417) 95%CI(-4.996, 0.52), t=-1.621, p=0.106	-0.971 (SE=1.582) 95%CI(-4.35, 2.346), t=-0.614, p=0.539	4.21 (SE=0.793) 95%CI(2.496, 5.643), t=5.308, p=<0.001
Group (left):T4	0.044 (SE=0.351) 95%CI(-0.798, 0.642), t=0.125, p=0.901	6.531 (SE=2.56) 95%CI(2.01, 12.568), t=2.551, p=0.011	-1.374 (SE=1.417) 95%CI(-4.154, 1.334), t=-0.969, p=0.333	0.307 (SE=1.582) 95%CI(-2.347, 2.65), t=0.194, p=0.846	0.586 (SE=0.793) 95%CI(-1.33, 1.931), t=0.739, p=0.461
Group (left):T5	0.015 (SE=0.351) 95%CI(-0.806, 0.87), t=0.043, p=0.965	-10.179 (SE=2.56) 95%CI(-15.117, -4.452), t=-3.975, p=<0.001	-5.855 (SE=1.417) 95%CI(-8.806, -3.038), t=-4.13, p=<0.001	-3.662 (SE=1.582) 95%CI(-7.101, -0.494), t=-2.315, p=0.021	4.248 (SE=0.793) 95%CI(2.361, 5.907), t=5.356, p=<0.001
Group (left):T6	-0.354 (SE=0.351) 95%CI(-0.989, 0.331), t=-1.009, p=0.314	-8.293 (SE=2.56) 95%CI(-13.103, -3.456), t=-3.239, p=0.001	-2.269 (SE=1.417) 95%CI(-4.935, 0.043), t=-1.601, p=0.11	1.948 (SE=1.582) 95%CI(-1.052, 4.921), t=1.231, p=0.219	4.905 (SE=0.793) 95%CI(3.282, 6.386), t=6.185, p=<0.001
Group (left):T7	0.131 (SE=0.351) 95%CI(-0.58, 0.964), t=0.374, p=0.708	1.298 (SE=2.56) 95%CI(-4.499, 6.447), t=0.507, p=0.613	0.174 (SE=1.417) 95%CI(-2.653, 2.88), t=0.123, p=0.902	2.769 (SE=1.582) 95%CI(-0.393, 5.685), t=1.751, p=0.081	0.638 (SE=0.793) 95%CI(-0.929, 2.252), t=0.805, p=0.421
Group (left):T8	0.125 (SE=0.351) 95%CI(-0.752, 0.745), t=0.357, p=0.722	3.607 (SE=2.56) 95%CI(-1.145, 8.933), t=1.409, p=0.16	-2.024 (SE=1.417) 95%CI(-4.909, 0.523), t=-1.428, p=0.154	0.024 (SE=1.582) 95%CI(-2.994, 2.921), t=0.015, p=0.988	3.838 (SE=0.793) 95%CI(1.884, 5.743), t=4.84, p=<0.001
Group (left):T9	0.044 (SE=0.351) 95%CI(-0.69, 0.896), t=0.125, p=0.901	-0.84 (SE=2.56) 95%CI(-5.655, 4.569), t=-0.328, p=0.743	1.021 (SE=1.417) 95%CI(-1.897, 3.568), t=0.721, p=0.472	6.486 (SE=1.582) 95%CI(3.242, 10.612), t=4.101, p=<0.001	4.371 (SE=0.793) 95%CI(2.012, 6.036), t=5.513, p=<0.001

Group (left):T10	0.049 (SE=0.351) 95%CI(-0.795, 0.773), t=0.138, p=0.89	1.312 (SE=2.56) 95%CI(-4.447, 6.455), t=0.512, p=0.609	2.388 (SE=1.417) 95%CI(-0.308, 4.886), t=1.685, p=0.093	5.933 (SE=1.582) 95%CI(2.455, 8.734), t=3.752, p=<0.001	4.371 (SE=0.793) 95%CI(2.581, 5.543), t=5.513, p=<0.001

Group (left): Result comparing left leg against right leg as reference. (SE): model coefficient (standard error); 95%CI: 95% confidence interval; t: Significant if p<0.05 (shown in red).

Supplementary material. Table 5: Radiofrequency parameters between both legs model pairwise results.

	Frequency (Hz)	Maximum power (Watts)	Average power (Watts)	Absorved energy (Volt-ampere)	Temperature (Celsius)
Right-T2 vs Left-T2	0.14 (SE=0.25) 95%CI(-0.766, 1.045), t=0.558, p=>0.999	-0.756 (SE=1.863) 95%CI(-7.506, 5.994), t=-0.406, p=>0.999	-1.654 (SE=1.011) 95%CI(-5.315, 2.008), t=-1.635, p=>0.999	1.766 (SE=1.149) 95%CI(-2.398, 5.93), t=1.536, p=>0.999	3.354 (SE=0.575) 95%CI(1.271, 5.437), t=5.834, p=<0.001
Right-T3 vs Left-T3	-0.006 (SE=0.25) 95%CI(-0.912, 0.9), t=-0.024, p=>0.999	2.694 (SE=1.863) 95%CI(-4.056, 9.444), t=1.446, p=>0.999	0.644 (SE=1.011) 95%CI(-3.018, 4.306), t=0.637, p=>0.999	2.737 (SE=1.149) 95%CI(-1.427, 6.901), t=2.382, p=>0.999	-0.855 (SE=0.575) 95%CI(-2.938, 1.228), t=-1.488, p=>0.999
Right-T4 vs Left-T4	0.096 (SE=0.25) 95%CI(-0.81, 1.002), t=0.383, p=>0.999	-7.287 (SE=1.863) 95%CI(-14.037, -0.537), t=-3.911, p=0.016	-0.28 (SE=1.011) 95%CI(-3.941, 3.382), t=-0.277, p=>0.999	1.458 (SE=1.149) 95%CI(-2.705, 5.622), t=1.269, p=>0.999	2.768 (SE=0.575) 95%CI(0.685, 4.851), t=4.815, p=<0.001
Right-T5 vs Left-T5	0.124 (SE=0.25) 95%CI(-0.781, 1.03), t=0.497, p=>0.999	9.423 (SE=1.863) 95%CI(2.673, 16.173), t=5.058, p=<0.001	4.201 (SE=1.011) 95%CI(0.54, 7.863), t=4.155, p=0.006	5.428 (SE=1.149) 95%CI(1.264, 9.591), t=4.723, p=<0.001	-0.893 (SE=0.575) 95%CI(-2.976, 1.19), t=-1.554, p=>0.999
Right-T6 vs Left-T6	0.494 (SE=0.25) 95%CI(-0.412, 1.4), t=1.975, p=>0.999	7.537 (SE=1.863) 95%CI(0.787, 14.287), t=4.046, p=0.01	0.616 (SE=1.011) 95%CI(-3.046, 4.277), t=0.609, p=>0.999	-0.182 (SE=1.149) 95%CI(-4.346, 3.982), t=-0.158, p=>0.999	-1.551 (SE=0.575) 95%CI(-3.634, 0.532), t=-2.697, p=>0.999
Right-T7 vs Left-T7	0.008 (SE=0.25) 95%CI(-0.898, 0.914), t=0.033, p=>0.999	-2.053 (SE=1.863) 95%CI(-8.804, 4.697), t=-1.102, p=>0.999	-1.827 (SE=1.011) 95%CI(-5.489, 1.834), t=-1.807, p=>0.999	-1.003 (SE=1.149) 95%CI(-5.167, 3.16), t=-0.873, p=>0.999	2.716 (SE=0.575) 95%CI(0.633, 4.799), t=4.724, p=<0.001
Right-T8 vs Left-T8	0.014 (SE=0.25) 95%CI(-0.891, 0.92), t=0.057, p=>0.999	-4.363 (SE=1.863) 95%CI(-11.113, 2.387), t=-2.342, p=>0.999	0.37 (SE=1.011) 95%CI(-3.291, 4.032), t=0.366, p=>0.999	1.742 (SE=1.149) 95%CI(-2.422, 5.906), t=1.516, p=>0.999	-0.484 (SE=0.575) 95%CI(-2.567, 1.599), t=-0.842, p=>0.999

Right-T9 vs Left-T9	0.096 (SE=0.25) 95%CI(-0.81, 1.002), t=0.383, p=>0.999	0.085 (SE=1.863) 95%CI(-6.666, 6.835), t=0.045, p=>0.999	-2.675 (SE=1.011) 95%CI(-6.337, 0.987), t=-2.646, p=>0.999	-4.72 (SE=1.149) 95%CI(-8.884, -0.556), t=-4.107, p=0.007	-1.017 (SE=0.575) 95%CI(-3.1, 1.066), t=-1.769, p=>0.999
Right-T10 vs Left-T10	0.091 (SE=0.25) 95%CI(-0.815, 0.997), t=0.364, p=>0.999	-2.068 (SE=1.863) 95%CI(-8.818, 4.683), t=-1.11, p=>0.999	-4.042 (SE=1.011) 95%CI(-7.703, -0.38), t=-3.998, p=0.011	-4.168 (SE=1.149) 95%CI(-8.332, -0.004), t=-3.626, p=0.049	-1.017 (SE=0.575) 95%CI(-3.1, 1.066), t=-1.769, p=>0.999

(SE): model coefficient (standard error); 95%CI: 95% confidence interval. Significant if $p<0.05$ (shown in red).
